# The Impact of Weight Loss on the Physiological Endotypes Associated With OSA

**DOI:** 10.1016/j.chest.2026.02.003

**Published:** 2026-02-17

**Authors:** Caroline J. Beatty, Ai-Ming Wong, Shane A. Landry, Luke D. J. Thomson, Jinny Collet, Veronica Odeke, Simon A. Joosten, Julie Playfair, Atul Malhotra, Kirk Kee, Matthew T. Naughton, Kate Sutherland, Peter A. Cistulli, Sanjay R. Patel, Wendy A. Brown, Garun S. Hamilton, Bradley A. Edwards

**Affiliations:** Department of Physiology (C. J. B., S. A. L., L. D. J. T., J. C., V. O., and B. A. E.), Biomedicine Discovery Institute, School of Psychological Sciences and Turner Institute for Brain and Mental Health (C. J. B., S. A. L., L. D. J. T., J. C., V. O., and B. A. E.), School of Clinical Sciences (A.-M. W., S. A. J., G. S. H.), and School of Translational Medicine (K. K. and M. T. N.), Monash University; Monash University Department of Surgery (J. P. and W. A. B.), Alfred Health; Monash Lung, Sleep, Allergy and Immunology (A.-M. W., S. A. J., and G. S. H.), Monash Health; Department of Respiratory and Sleep Disorders Medicine (K. K.), The Royal Melbourne Hospital; Department of Respiratory Medicine (M. T. N.), Alfred Hospital, Melbourne; Sleep Research Group (K. S. and P. A. C.), Charles Perkins Centre, Faculty of Medicine and Health, University of Sydney, Camperdown; Department of Respiratory and Sleep Medicine (K. S. and P. A. C.), Royal North Shore Hospital, Sydney, NSW, Australia; Division of Pulmonary, Critical Care and Sleep Medicine (A. M.), University of California, San Diego (UCSD), CA; and Division of Pulmonary, Allergy, Critical Care and Sleep Medicine (S. R. P.), University of Pittsburgh, Pittsburgh, PA.

**Keywords:** endotypes, metabolic bariatric surgery, obstructive sleep apnea, pathophysiology, weight loss

## Abstract

**BACKGROUND:**

Weight loss improves upper airway collapsibility in people with OSA. However, it is unclear how weight loss affects the other physiological traits (ie, endotypes) associated with OSA (loop gain, arousal threshold, and muscle compensation).

**RESEARCH QUESTIONS:**

What is the effect of metabolic bariatric surgery on the OSA endotypes, and are the baseline endotypes associated with improvements in OSA severity?

**STUDY DESIGN AND METHODS:**

Using noninvasive methods, the OSA endotypes were measured during overnight clinical polysomnography in 43 patients before and after (approximately 6–18 months) metabolic bariatric surgery. OSA endotypes were also measured, using reference standard physiological techniques (ie, CPAP dial-down method) in a subset of patients (n = 10). Generalized linear mixed-effects models were used to evaluate the effect on outcome variables.

**RESULTS:**

Metabolic bariatric surgery was associated with improvements in apnea-hypopnea index (AHI; 50.7 ± 26.8 vs 24.6 ± 19.2 events/h; *P <* .001), BMI (42.1 ± 5.5 vs 32.8 ± 4.5 kg/m^2^; *P <* .001), upper airway collapsibility (67.0% ± 21.4% vs 72.8% ± 16.2% V_eupnea_; *P* = .011) and loop gain (0.65 ± 0.19 vs 0.58 ± 0.19; *P* = .011). Weight loss also reduced arousal threshold (149.7% ± 28.6% vs 142.6% ± 30.1% V_eupnea_; *P* = .011). Reference standard measures showed changes consistent with noninvasively derived endotypes. A greater improvement in airway collapsibility was associated with a greater reduction in AHI (*B* = −0.62; 95% CI, −0.97 to −0.27; *P* = .002; *R*^2^ = 0.23), and greater decreases in arousal threshold were associated with greater improvements in AHI (*B* = 0.63; 95% CI, 0.33−0.92; *P <* .001; *R*^2^ = 0.29). Multivariable models controlling for baseline AHI found that those with less-collapsible upper airways and low loop gain at baseline may be more likely to see improvements in their OSA severity after surgery.

**INTERPRETATION:**

Our results show that metabolic bariatric surgery is associated with improvements in weight, AHI, upper airway collapsibility, and loop gain and may be particularly effective for those with mild upper airway collapsibility and low loop gain.

Obesity is a global public health challenge, with nearly 2.5 billion adults worldwide considered overweight or obese.^1^ Having obesity is associated with numerous adverse health consequences, including OSA. OSA is a common sleep disorder, in which the upper airway collapses repeatedly throughout the night despite breathing efforts. Repeated collapse of the upper airway leads to disturbances in blood oxygenation and fragmented sleep, which can result in poor health outcomes such as hypertension and excessive daytime sleepiness. Obesity is the most important reversible risk factor for OSA, with an estimated 70% of people with OSA considered overweight or obese. ^2^ Because of the prevalence of both obesity and OSA, practical treatment options that address both conditions are warranted.

Obesity contributes to OSA by placing an additional load on the upper airway and abdomen, increasing the propensity to upper airway collapse. A pivotal study by Schwartz and colleagues ^3^ found that upper airway collapsibility improved in people with OSA following medical weight loss. As such, weight loss is recommended in conjunction with CPAP to treat OSA in people who are overweight. ^4^ However, both CPAP and weight loss come with challenges. CPAP is very effective in improving OSA, but it is not always well tolerated ^5^ and some evidence suggests that it may promote weight gain. ^6^

Because of the major challenges of losing weight and the adverse health effects of obesity, metabolic bariatric surgery (MBS) may be recommended for those with a BMI ≥ 30 kg/m^2^ with comorbidities.^7^ Notably, MBS is more effective than lifestyle interventions and has longer-lasting effects, with weight loss often sustained 10 years after surgery.^8^ Obesity management medications such as tirzepatide (GLP-1 and GIP agonist) and semaglutide (GLP-1 agonist) have shown promising weight loss results; however, continued treatment with obesity management medications is required for weight maintenance.^9, 10^ In contrast, MBS is usually a once-off procedure.

Importantly, although MBS has also been found to improve OSA severity, ^11^ the relationship between the amount of weight lost and the improvement in OSA severity is not strictly linear. ^12^ Despite successful weight loss, many who undergo MBS with OSA are still living with obesity and have clinically significant OSA. The variability in OSA resolution following weight loss suggests that other factors besides obesity may be involved in promoting upper airway collapse. ^13^ Indeed, besides having a highly collapsible upper airway, several other endotypes contribute to the development of OSA. These endotypes include the following: having (1) an oversensitive ventilatory control system, known as high loop gain; (2) a low respiratory arousal threshold; and (3) poor upper airway muscle compensation. ^14^ One or more of these endotypes may contribute to whether a person develops OSA, meaning not everyone develops OSA for the same reason. Therefore, this study aimed to measure OSA severity and the OSA endotypes before and after MBS and to determine if any of these baseline characteristics were associated with OSA resolution. We hypothesized that OSA resolution following weight-loss surgery is driven by improvements in several OSA traits, not simply just the anatomy/collapsibility.

## Study Design and Methods

The current study combined data from three independent studies: one prospective study (Predicting the successful resolution of Obstructive Sleep Apnoea following weight-loss surgery [BAROSA] trial ^15^ ), which was originally designed to answer our research questions (approved by the Alfred Health Ethics Committee [Project ID: 6225] and registered with the Monash University Ethics Committee [Project ID: 28299]; Australian New Zealand Clinical Trials Registry [ACTRN12619001505190]), and retrospective analysis of data from 2 published studies (Bakker and colleagues ^16^ and Sutherland and colleagues ^17^; consent to use these data sets was granted by the Monash University Human research ethics committee [Project IDs: 20346 and 14937]). See the Supplementary Methods in the online article for details regarding the rationale behind combining multiple data sets as well as the methods used in each of the trials.

### BAROSA trial:

The BAROSA trial was designed to assess the effect of MBS on the OSA endotypes. For this study, participants scheduled to undergo sleeve gastrectomy (SG) took part in producing an overnight attended laboratory polysomnogram (PSG) before and within 6 to 12 months after surgery.

### ABC (Apnea, Bariatric Surgery vs CPAP) trial (Bakker and colleagues^16^):

This was a randomized controlled trial comparing the management of OSA with CPAP vs MBS. Participants randomized to receive MBS had laparoscopic gastric banding. Participants underwent overnight attended polysomnography in the laboratory before and 18 months after surgery. Only data from participants who underwent surgery were included in this analysis.

### Sutherland and colleagues^17^:

The aim of this study was to establish the effect of MBS on the upper airway volume and soft tissues. Participants underwent either a SG or a one-anastomosis gastric bypass (OAGB) procedure. Participants produced an overnight attended laboratory PSG before and 6 months after surgery.

### Clinical Polysomnography

Polysomnography was performed in a similar manner across all trials, and included an EEG, chin electromyogram, electro-oculogram, ECG, respiratory effort, oxygen saturation, snoring, and body position. At each site, airflow was measured with a nasal pressure cannula and oronasal thermistor. Sleep state, arousal, and respiratory events were scored independently by each site, using American Academy of Sleep Medicine criteria. Hypopneas were scored using the “2012 recommended” definition^18^ (specifically, airflow signal reduced by ≥ 30% of baseline with ≥ 3% oxygen desaturation or cortical arousal). The raw PSG signals were collected and reviewed from all sites and exported into European Data Format, and then imported into Matlab (Math-Works) ^19^ for analysis of the OSA endotypes (airway collapsibility [per V_passive_ ], loop gain, arousal threshold, and muscle compensation), using previously validated and published methods.^20–22^ The OSA endotypes were quantified from non-rapid eye movement (NREM) sleep only, and estimates from NREM all positions (primary outcome) and NREM supine (sensitivity analyses) are reported.

### Research Polysomnography

In a subset of participants of the BAROSA trial, the OSA endotypes were also measured using the more invasive CPAP dial-down technique ^23^ during the production of 2 overnight research PSGs performed before and 6–12 months after surgery (both within 1–2 weeks of the clinical PSGs). The research PSG consisted of the same setup as the clinical PSG except that the nasal cannula and oronasal thermistor were replaced with a nasal mask with a pneumotachograph (model 3700A; Hans Rudolph) to measure ventilation. The nasal mask was attached to CPAP (Phillips-Respironics). Mask pressure was measured through a port in the nasal mask (Validyne), and O_2_/CO_2_ was recorded at the nostril via an O_2_/CO_2_ analyzer (VacuMed). The signals were sampled at 256 Hz and displayed using Spike2 software (Cambridge Electronic Design). Participants were then administered a therapeutic level of CPAP, and the CPAP levels were altered using previously validated and published techniques during supine NREM sleep to measure the OSA physiological endotypes.^23^

### Statistical Analysis

All data were analyzed with RStudio (R version 4.4.3; February 28, 2025). Upper airway collapsibility and arousal threshold values were transformed to improve normality.^24^ Generalized linear mixed-effects (GLME) models were used to evaluate the effect of visit (before vs after MBS) on outcome variables, accounting for repeated measures within participants and clustering by study source (eg, BAROSA, Bakker, or Sutherland). Models included visit as a fixed effect, and participant and study source were included as random intercepts (ie, outcome ∼ visit + (1|Study Source) + (1|Participant)). An identity link and Gaussian error distribution were used unless otherwise specified. GLME models were also applied to examine (1) associations between changes in OSA endotypes and ΔAHI (as well as ΔBMI), (2) associations between baseline endotypes and ΔAHI, and (3) the combined effect of baseline endotypes and AHI on ΔAHI, with random intercepts for study source to account for clustering of participants. All model assumptions were assessed by inspecting residual diagnostics, including normality of residuals (via histogram, Q-Q plot, and Shapiro-Wilk test), homoscedasticity (residuals vs fitted values), and model fit. Given our a priori focus on the four primary endotype changes following MBS, the Benjamini-Hochberg adjustment was applied to the *P* values for these primary hypotheses only. For these analyses, adjusted *P* values less than or equal to .05 were considered statistically significant. All additional analyses were considered exploratory and no adjustment for multiplicity was applied; unadjusted *P* values are reported for these analyses.

## Results

**Figure 1 –:**
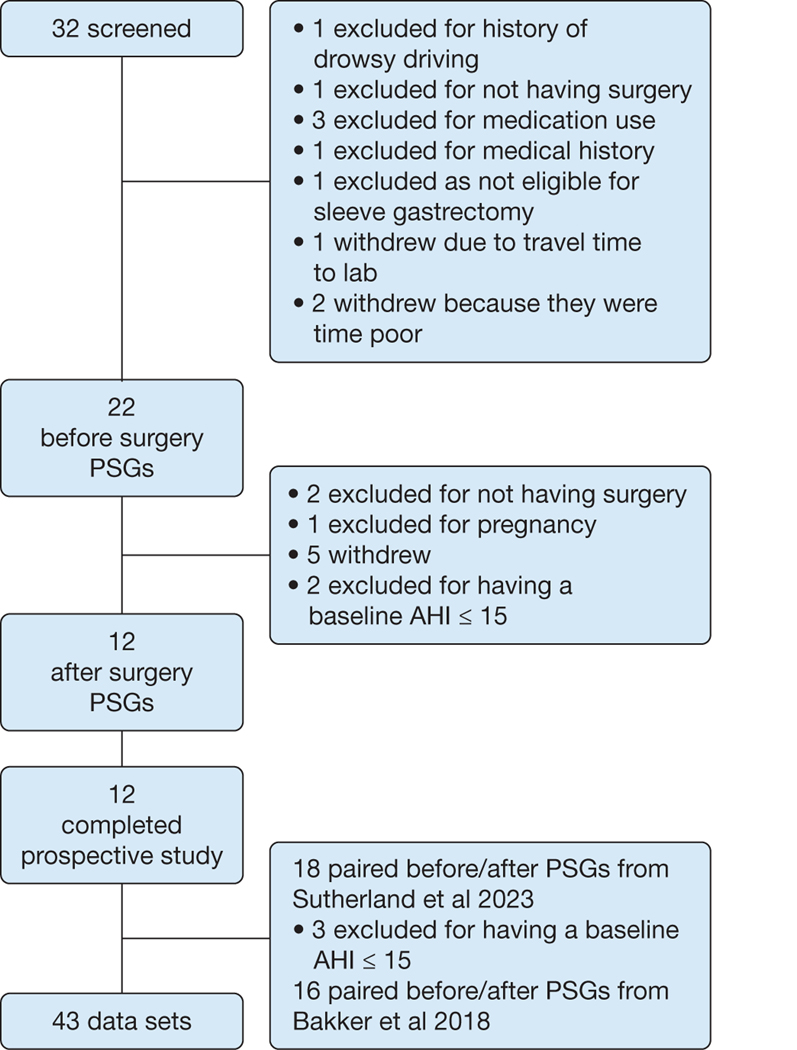
Flow chart of recruitment, exclusions, and withdrawals in the original prospective study, and the addition of retrospective data sets from Bakker and colleagues ^16^ and Sutherland and colleagues. ^17^ AHI = apnea-hypopnea index; PSG = polysomnogram.

Data were prospectively collected from 12 BAROSA trial participants and combined with retrospective data from 16 Bakker trial participants and 15 Sutherland trial participants; therefore, data from a total of 43 participants were analyzed (Fig 1). In total, 3 participants were excluded from analysis due to an AHI < 15 events/h at baseline. In total, 24 participants (55.8%) had an SG, 4 (9.3%) had an OAGB, and 15 (34.9%) had laparoscopic gastric banding. Most participants were living with class 3 obesity (58%; BMI, > 40 kg/m^2^) and had severe OSA (70%; AHI, ≥ 30 events/h). Participant baseline characteristics can be found in Table 1.

### Anthropometric and Sleep Characteristics

**Figure 2 –:**
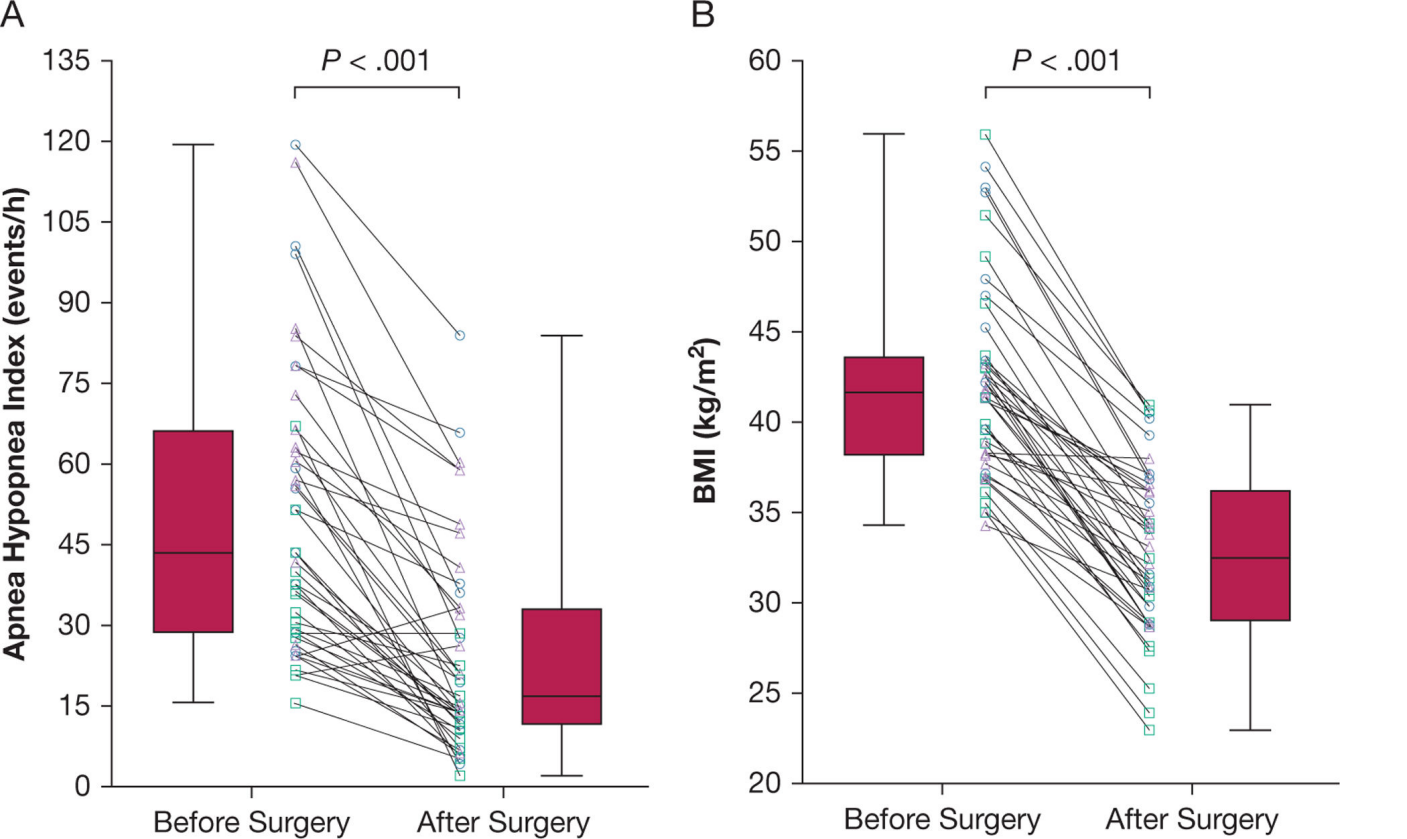
A, Boxplots showing OSA severity as measured by the apnea-hypopnea index (AHI) before and after metabolic bariatric surgery (MBS). There was a significant reduction in AHI following MBS. However, the line series graph shows considerable individual differences, with most participants having improved OSA, but some who worsened or stayed the same. Furthermore, after surgery most people still have clinically significant OSA requiring treatment. B, Boxplots showing BMI before and after MBS. There was a significant reduction in BMI before and after MBS. The line series shows considerable individual differences in BMI reduction. Despite significant weight loss, after surgery most participants are still categorized as overweight or obese. Blue circles = BAROSA trial, purple triangles = ABC trial, green squares = Sutherland trial.

The anthropometric and sleep characteristics before and after MBS are presented in Table 2. Participants lost a significant amount of weight (−27.0 [–30.0 to –23.3] kg; *P <* .001), which was reflected by significant improvements in BMI (−9.3 [−10.6 to –8.1] kg/m^2^; *P <* .001). In addition, participants improved their AHI (−26.1 [–32.0 to –20.3] events/h; *P <* .001) (Fig 2) and subjective sleepiness (Epworth Sleepiness Scale, −3.1 [−4.5 to −1.7]). In total, 2 participants (4.7%) had a postoperative AHI < 5 events/h. Overall, there was a 21.8% ± 8.7% reduction in weight. Furthermore, a GLME model demonstrated that the percentage BMI reduction was significantly associated with percentage 4 improvement in AHI (standardized β = 1.69, *P* = .0004, marginal *R*^2^ = 0.257), indicating that greater weight loss was associated with greater reductions in OSA severity. Comparisons between baseline and change characteristics between trials are reported in the online article (e-Table 1).

### OSA Endotypes

The impact of MBS on the OSA endotypes (for both NREM all positions and NREM supine) is detailed in Table 3.^25, 26^ When the OSA endotypes were measured during NREM all positions (primary outcome, N = 43), upper airway collapsibility (8.7 [3.0 to 14.4]% V_eupnea_; *P* = .011) and loop gain (−0.07 [−0.11 to −0.02]; *P* = .011) were improved following MBS. The arousal threshold was also reduced (−7.1 [−12.3 to −1.9]% V_eupnea_; *P* = .011), but there was no significant change in muscle compensation. These findings were not impacted by sex (e-Table 2) and were mirrored when the endotypes were derived from the research polysomnography (e-Fig 1, e-Tables 3 and 4). Monthly rates of change in the OSA endotypes (as well as AHI, weight, and BMI) are provided in the supplemental material (e-Table 5). When our analyses were restricted to the NREM supine position (n = 33), although the estimates were of a similar magnitude, only collapsibility was improved following MBS after multiplicity adjustment. There were no significant associations between the change in BMI and change in any of the OSA endotypes. Furthermore, there was no significant difference in the change in any of the OSA endotypes between the 3 different trials (e-Table 1).

### Factors Associated With OSA Improvement

GLME models demonstrated that changes in upper airway collapsibility and arousal threshold were significantly associated with changes in AHI (Table 4). A greater improvement in airway collapsibility was associated with a greater reduction in AHI (unstandardized *B* = −0.62; 95% CI, −0.97 to −0.27; *P* = .002; *R*^2^ = 0.23). Similarly, greater decreases in arousal threshold were associated with greater improvements in AHI (unstandardized *B* = 0.63; 95% CI, 0.33–0.92; *P <* .001; *R*^2^ = 0.29). However, changes in loop gain and muscle compensation were not related to the change in AHI.

### Baseline Associations

Univariable GLME models were then used to assess whether the baseline OSA endotypes were associated with change in AHI after MBS (Table 4). Specifically, a more collapsible airway at baseline tended to be associated with greater reductions in AHI (unstandardized *B* = 0.33; 95% CI, 0.07–0.60; *P* = .060; *R*^2^ = 0.13), although this effect did not reach statistical significance after correction for multiple comparisons. Baseline loop gain, arousal threshold, and muscle compensation were not associated with the change in AHI (all *P >* .16).

In multivariable models including baseline AHI and baseline OSA endotypes, the pattern of associations with ΔAHI shifted compared with univariable analyses (Table 5). In model 1, which included baseline airway collapsibility as a significant univariable association, participants with milder airway collapsibility (ie, less collapsible/stiffer airway) at baseline experienced greater reductions in AHI (standardized β = −0.58; *P <* .001), indicating that after accounting for baseline disease severity, milder collapsibility was associated with greater improvement. In model 2, baseline loop gain was added given its association with outcomes of other OSA therapies. Here, lower loop gain emerged as an additional characteristic associated with ΔAHI (standardized β = 0.23; *P* = .040). In model 3, incorporating all OSA endotypes, airway collapsibility and loop gain remained associated with the outcome (ie, similar β estimates), although the association with loop gain was no longer statistically significant. Similar findings were observed when participants were categorized as responders vs nonresponders using either (1) a > 50% reduction in AHI with after MBS AHI < 15 events/h, or (2) after MBS AHI < 15 events/h alone (see e-Tables 6 and 7).

## Discussion

This study aimed to measure the impact of weight loss following MBS on OSA severity and the underlying OSA endotypes. Our findings suggest weight loss following MBS improves OSA severity, upper airway collapsibility, and loop gain. However, following MBS, the arousal threshold decreases (making people more arousable). Furthermore, after controlling for baseline OSA severity, those with less collapsible upper airways as well as low loop gain presurgery may be more likely to see improvements in their OSA severity after surgery.

The finding that surgically induced weight loss resulted in significant weight reduction and reduction in OSA severity is consistent with previous research.^11, 27,28^ Furthermore, the degree of weight loss achieved between 6 and 18 months (−21.8% of total body weight) is similar to the reported weight loss in other studies of MBS for OSA,^11^ and to a study using the novel dual GLP-1/GIP agonist tirzepatide (approximately 18%−20% reduction in body weight at 52 weeks).^29^ However, most participants in this study and in the previous research were still living with obesity after surgery and still had clinically significant OSA at the time of follow-up.

Consistent with previous findings, ^3^ the current study demonstrated that upper airway collapsibility improved with weight loss irrespective of whether it was measured noninvasively (from the PSG signals) or invasively. Indeed, our findings reflect the findings from cross-sectional studies reporting a relationship between increasing obesity levels and progressively worsening upper airway collapsibility. ^30,31^ Although this is the first study to noninvasively quantify changes in collapsibility with weight loss, the improvement (8.7%−11.8% V_eupnea_) was smaller than that observed with mandibular advancement devices (approximately 22%)^32^ or upper-airway surgery (approximately 40%),^33^ likely due to more severe baseline collapsibility in those cohorts (see e-Table 8). In contrast, our invasively measured improvements (3.9 L/min) are similar to those reported previously,^3^ and exceeded values reported for mandibular advancement (2.8 L/min),^34^ lateral positioning (approximately 3.3 L/min), ^35^ and surgery (approximately 1.9 L/min) ^33^ despite comparable baseline severity. Overall, the magnitude of improvement in upper airway collapsibility with weight loss appears both method and cohort (ie, baseline characteristics) dependent. Nonetheless, although the improvement in collapsibility was modest, it correlated moderately with AHI reduction (*R*^2^ = 0.23) and independently predicted treatment response. Given the ongoing uncertainty about the magnitude of trait change required for clinically meaningful benefit,^26, 36^ this association is promising and supports collapsibility as a relevant mechanistic target of weight-loss therapy.

Another key finding of our study was that weight loss via MBS reduced loop gain (sensitivity of the ventilatory control system). These findings mirror the cross-sectional data from Sands and colleagues^31^ showing that increased BMI is associated with increased loop gain. Although we cannot derive the cause of the reductions in loop gain, several potential mechanisms have previously been proposed, including changes in lung volume or alterations in leptin.^37^ However, our study found no association between weight loss and the change in loop gain (see e-Fig 2), suggesting that the reduction in loop gain may not be due to weight loss specifically but was perhaps a consequence of the reduction in AHI. Notably, previous studies have found a decrease in loop gain and AHI with upper airway surgery, ^33,38,39^ which showed no change in weight. Furthermore, the scale of change in both loop gain and AHI in this study is relatively consistent with those found in the previous upper airway surgery studies. ^33,38,39^ Collectively, such evidence supports the hypothesis that an elevated loop gain may be a consequence of OSA (see the supplemental material, Section 7 [e-Fig 3 and e-Table 9], for more detail).

The current study also demonstrated that MBS reduced the arousal threshold (made people more arousable). The reason for this reduction is currently unclear, but previous studies have found similar reductions in arousal threshold with CPAP treatment ^40,41^ and upper airway surgery, ^33,38,42^ similar to loop gain. Interestingly, a meta-analysis employed meta-regression equations to predict changes in the respiratory arousal threshold corresponding to a given change in AHI.^29, 43^ When this equation was fitted to the current data, the observed change in arousal threshold was smaller than would be expected based on the meta-regression equation, suggesting that the changes observed in the current study are largely explained by reductions in the AHI. Taken together, these findings suggest that arousal threshold may increase as a consequence of OSA and that treating the OSA leads to a subsequent reduction. However, further research into the direct mechanisms by which both loop gain and arousal threshold were reduced in the current study and others is warranted.

Although we observed that the degree of weight loss was associated with the degree of improvement in the AHI, and that certain endotype changes were associated with the change in AHI, we did not find a direct association between the change in weight and the changes in the endotypes. This may reflect limited statistical power, measurement noise in the endotype estimates, or heterogeneous pathways by which weight loss improves OSA severity. ^44^ Crucially, the absence of a significant delta weight to delta endotype relationship in this cohort does not preclude a mediating role of endotypes, but underscores the need for more precise methods of assessing endotypes, larger studies with repeated endotype assessments, and more sensitivity to assess these indirect pathways.

The current results also suggest that mild collapsibility and low loop gain at baseline were key endotypes associated with improvements in OSA severity following MBS (after controlling for baseline OSA severity). Although baseline AHI emerged as the strongest predictor of postoperative improvements in OSA severity, this association should be interpreted with caution. Because of measurement error, people with more severe OSA at baseline will tend to have lower AHI on repeat assessment even without any intervention, and part of the observed relationship may therefore reflect regression to the mean. This phenomenon limits the clinical interpretability of baseline AHI as a prognostic marker. Importantly, our multivariable models therefore included baseline AHI to prevent this statistical effect from obscuring the independent contributions of the physiological endotypes. There is now an established body of literature that supports the use of OSA endotypes in helping to understand response to OSA therapies, ^24,34,38,45^ including interventions targeting upper airway anatomy. For example, oral appliance studies have shown that participants with mild to moderate collapsibility tend to have a greater reduction in AHI.^34, 45,46^ Importantly, identifying mild collapsibility and low loop gain as indicators of treatment response to MBS provides valuable information beyond baseline OSA severity. ^45^

This study had several strengths and limitations that must be considered. A major strength of our analyses was that we controlled for the between site/surgery differences statistically by including them as random effects in our models. Another strength of the study was that the OSA endotypes were also measured using the more invasive methods in a subset of the data from the original study. Similar to the original validation studies,^20–22^ the noninvasive estimates were moderately correlated with the invasive measures (e-Fig 4) and demonstrated consistent relative effect sizes across methods (e-Table 10). Despite the small subgroup analysis, the impact of weight loss on the OSA endotypes was nearly identical irrespective of the method used to measure them. Limitations of our study were that we did not have a control group to compare our changes against and were unable to explore whether body position or craniofacial structure may explain the variability in OSA response to weight loss. Given that we only studied the impact of weight loss, not weight gain, we were not able to assess whether certain endotypes (such as strong upper airway muscle responsiveness) protect against the development of obesity-related OSA in the first place.^44, 47^ Moreover, because of our relatively small sample size, we were unable to perform formal mediation analyses to determine the extent to which changes in endotypes mediate the relationship between weight loss (ΔBMI) and improvements in OSA severity (ΔAHI). Well-powered mediation analyses in larger data sets (eg, A Study of Tirzepatide in Participants With Obstructive Sleep Apnea [SURMOUNT-OSA] trial^29^) are needed to clarify how much of the effect of weight loss on AHI is explained by changes in OSA endotypes.

### Interpretation

This study provides insights into how obesity contributes to OSA. Our findings suggest that obesity predominantly affects upper airway collapsibility. Whether or not the reductions in loop gain and arousal threshold observed in this study were directly caused by reduction in weight following MBS or were a consequence of the improvement in OSA severity requires further investigation. Future work should focus on confirming our findings in larger cohorts (in response to different methods of weight loss) as well as on identifying how much weight loss is required to see improvements in the OSA endotypes. As one of the goals of the current study was to attempt to help guide clinicians on which patients might benefit the most from surgical weight loss, our results suggest that those with mild upper airway collapsibility and low loop gain may benefit the most after MBS.

## Supplementary Material

sup

**Additional information:** The e-Figures, eTables, and Supplementary Methods are available online under “Supplementary Data.”

## Figures and Tables

**TABLE 1 ]:** Baseline Participant Characteristics

Characteristic	N = 43

Age, y	47.2 ± 10.0
Sex (male)	18 (41.9)
Weight, kg	122.8 ± 22.3
BMI, kg/m^2^	41.6 [38.2, 43.5]
AHI, events/h	43.4 [28.6, 64.8]
Surgery type	
Laparoscopic gastric banding	15 (34.9)
One-anastomosis gastric bypass	4 (9.3)
Sleeve gastrectomy	24 (55.8)

Data are presented as Mean ± SD, No. (%), or median [Q1, Q3] unless otherwise indicated. AHI = apnea-hypopnea index.

**TABLE 2 ]:** Anthropometric and Sleep Characteristics Before and After Metabolic Bariatric Surgery

Characteristic	Baseline^a^	Follow-Up ^a^	Difference Estimate (95% CI)	*P* Value

Anthropometric measurements				
Weight, kg	122.8 ± 22.3	95.8 ± 18.7	–27.0 (–30.0 to –23.3)	< .001
BMI, kg/m^2^	42.1 ± 5.5	32.8 ± 4.5	−9.3 (−10.6 to –8.1)	< .001
Epworth Sleepiness Scale ^b^	8.6 ± 3.5	5.6 ± 3.0	−3.1 (−4.4 to −1.7)	< .001
Sleep characteristics				
TST, min	383.0 [336.0–426.5]	402.5 [362.2–443.0]	33.4 (6.6 to 60.2)	.015
WASO, min	60.5 [41.5–86.8]	44.5 [29.5–66.0]	–23.8 (–44.7 to −3.0)	.026
N1 sleep duration, min	60.0 [27.0–80.3]	40.5 [31.5–50.3]	−17.4 (−28.0 to −6.9)	.002
N2 sleep duration, min	196.7 ± 56.3	223.8 ± 56.9	27.1 (6.1 to 48.1)	.012
N3 sleep duration, min	53.0 [12.2–84.8]	50.0 [28.8–73.8]	2.3 (−9.2 to 13.9)	.700
NREM sleep duration, min	330.5 [287.5–352.5]	335.0 [294.0–356.0]	11.9 (−9.4 to 33.3)	.270
REM sleep duration, min	57.0 [33.6–77.9]	72.5 [54.0–99.4]	21.5 (11.6 to 31.4)	< .001
AHI total, events/h	50.7 ± 26.8	24.6 ± 19.2	–26.1 (–32.0 to –20.3)	< .001
AHI NREM, events/h	47.3 ± 28.8	22.5 ± 21.2	–24.8 (–30.7 to −18.9)	< .001
AHI REM, events/h	65.4 ± 27.2	31.1 ± 22.0	–34.1 (–42.3 to –25.8)	< .001
AHI supine, events/h	62.1 ± 33.9	28.2 ± 24.6	–34.0 (–44.1 to –24.0)	< .001
AHI NREM supine, events/h	55.9 ± 38.1	26.1 ± 26.3	–29.8 (–40.6 to −19.0)	< .001
Respiratory Arousal Index, events/h	33.7 [27.0–52.6]	22.1 [16.1–32.1]	−15.1 (−20.4 to −9.9)	< .001
F_Hypopneas_	0.9 [0.7–0.9]	0.9 [0.8–1.0]	0.07 (0.02 to 0.13)	.015
Spo_2_ nadir (%)	80.0 [72.5–84.0]	84.0 [81.0–87.0]	6.2 (3.5 to 8.9)	< .001
SpO_2_ mean	93.7 [92.2–94.8]	94.8 [93.9–95.9]	1.9 (1.1 to 2.7)	< .001
ODI 3%, events/h	36.1 [23.6–54.8]	14.8 [7.1–29.8]	–22.7 (−27.1 to −18.2)	< .001
ODI 4%, events/h	26.8 [15.4–46.4]	8.5 [3.8–22.4]	–20.0 (−24.8 to −15.1)	< .001
Hypoxic burden, %min/h	83.7 [45.6–134.5]	29.6 [11.1–69.2]	–80.8 (−117.3 to –44.3)	< .001

AHI = apnea-hypopnea index; F_Hypopneas_ = fraction of respiratory events that were hypopneas; N1 = stage 1 sleep; N2 = stage 2 sleep; N3 = stage 3 sleep; NREM = non-rapid eye movement sleep; ODI = oxygen desaturation index; REM = rapid eye movement sleep; SpO_2_ = pulse oximeter oxygen saturation; TST = total sleep time; WASO = wake after sleep onset.

a Mean ± SD; median [interquartile range] depending on whether the model residuals were normally distributed. Difference estimate (unstandardized *B* and 95% CI) using generalized linear mixed-effects model of the order: Anthropometric/Sleep variable ∼ Visit + (1|Study Source) + (1|Participant).

b Subset of n = 28 for whom the Epworth Sleepiness Scale score was collected.

**TABLE 3 ]:** OSA Physiological Endotypes Before and After Metabolic Bariatric Surgery

Characteristic	Baseline Mean ± SD	Follow-Up Mean ± SD	Difference Estimate (95% CI)	Unadjusted *P* Value	Adjusted *P* Value

NREM all positions (N = 43)					
Upper airway collapsibility (% V_eupnea_) ^a^	67.0 ± 21.4	72.8 ± 16.2	8.7 (3.0 to 14.4)	.003	.011
Loop gain	0.65 ± 0.19	0.58 ± 0.19	−0.07 (−0.11 to −0.02)	.006	.011
Arousal threshold (% V_eupnea_)	149.7 ± 28.6	142.6 ± 30.1	−7.1 (−12.3 to −1.9)	.008	.011
Muscle compensation (% V_eupnea_ )	3.6 ± 13.9	−0.7 ± 18.0	−4.3 (−9.6 to 1.1)	.118	.118
NREM supine (n = 33)					
Upper airway collapsibility (% V_eupnea_)^a^	59.2 ± 23.6	68.7 ± 19.9	11.8 (5.5 to 18.0)	< .001	.004
Loop gain	0.65 ± 0.21	0.59 ± 0.25	−0.06 (−0.12 to −0.001)	.048	.079
Arousal threshold (% V_eupnea_)	151.5 ± 31.1	149.3 ± 33.0	−6.4 (−13.0 to 0.3)	.059	.079
Muscle compensation (% V_eupnea_)	2.3 ± 15.4	1.8 ± 15.7	−0.5 (−7.5 to 6.5)	.889	.889

Data shown in Baseline and Follow-Up columns represent mean ± SD. Difference estimate (unstandardized *B* and 95% CI) using generalized linear mixed-effects model of the order: Endotype ∼ Visit + (1|Study Source) + (1|Participant). *P* values were adjusted using the Benjamini-Hochberg correction to control for multiple comparisons of our main outcomes. When analyses were repeated with follow-up duration (months) included as an additional fixed-effect covariate, its inclusion did not alter the magnitude, direction, or statistical significance of any reported associations and was itself nonsignificant (all *P >* .3). NREM = non-rapid eye movement sleep.

a A custom link function was used in this model (similar to a logit link), allowing the mean–response relationship to be modeled after correction for previously described floor and ceiling effects of the endotype as described previously. ^25,26^

**TABLE 4 ]:** Individual Generalized Linear Mixed-Effects Models Exploring the Association Between the Change in Overall AHI vs the Change in the OSA Endotypes as Well as the Baseline OSA Endotypes

Model Predictor	Relationship to ΔAHI_total_ (events/h)						
	*B*	SE	95% CI	β	Unadjusted *P* Value	Adjusted *P* Value	Marginal *R*^2^

Change in the OSA endotypes							
Δ Upper airway collapsibility (% V_eupnea_)	−0.62	0.17	−0.97 to −0.27	−0.48	.0009	.0018	0.231
Δ Loop gain	20.60	18.79	−17.35 to 58.56	0.16	.279	.372	0.027
Δ Arousal threshold (% V_eupnea_)	0.63	0.15	0.33 to 0.92	0.55	.00009	.00036	0.294
Δ Muscle compensation (% V_eupnea_)	−0.04	0.17	−0.37 to 0.30	−0.04	.824	.824	0.001
Baseline OSA endotypes							
Upper airway collapsibility (% V_eupnea_)	0.33	0.13	0.07 to 0.60	0.36	.015	.060	0.129
Loop gain	−10.69	15.72	–42.45 to 21.07	−0.10	.500	.535	0.011
Arousal threshold (% V_eupnea_)	−0.18	0.10	−0.38 to 0.02	−0.26	.080	.160	0.07
Muscle compensation (% V_eupnea_ )	−0.13	0.21	−0.56 to 0.30	−0.09	.535	.535	0.009

Generalized linear mixed-effects models of the order: ΔAHI ∼ Δ(or Baseline) OSA Endotype + (1|Study Source). When analyses were repeated with follow-up duration (months) included as an additional fixed-effect covariate, its inclusion did not alter the magnitude, direction, or statistical significance of any reported associations and was itself nonsignificant (all *P >* .3). P values were adjusted using the Benjamini-Hochberg correction to control for multiple comparisons of our main outcomes. B = unstandardized β; SE = standard error of the estimated coefficient; β = standardized β; marginal *R*^2^ = proportion of total variance explained by the fixed effects only.

**TABLE 5 ]:** Multivariable GLME Models Showing the Baseline Characteristics Associated With Change in AHI With Metabolic Bariatric Surgery

Baseline Characteristic	Model l^a^ Marginal *R*^2^ = 0.63 Adjusted *R*^2^ = 0.61				Model 2^a^ Marginal *R*^2^ = 0.67 Adjusted *R*^2^ = 0.64				Model 3^a^ Marginal *R*^2^ = 0.69 Adjusted *R*^2^ = 0.65			
	*B*	SE	β	*P* Value	*B*	SE	β	*P* Value	*B*	SE	β	*P* Value

AHI, events/h	−0.87	0.11	−1.20	< .001	−0.91	0.11	−1.25	< .001	−0.90	0.11	−1.24	< .001
Upper airway collapsibi1ity,	−0.53	0.14	−0.58	< .001	−0.46	0.14	−0.50	.003	−0.33	0.16	−0.36	.045
% V_eupnea_												
Loop gain					23.5	11.1	0.23	.040	23.4	12.4	0.23	.068
Arousal threshold, % V_eupnea_									0.10	0.11	0.14	.358
Muscle compensation,									−0.16	0.14	−0.12	.240
% V_eupnea_												

Generalized linear mixed-effects model of the order: ΔAHI ~ Baseline AHI + Baseline Endotypes (added sequentially) + (1 |Study Source). β = standardized β; AHI = apnea-hypopnea index; *B* = unstandardized β; GLME = generalized linear mixed effects; SE = standard error of the estimated coefficient; marginal *R*^2^ = proportion of total variance explained by the fixed effects only.

a Note that adding age and BMI into all models as fixed effects did not impact the interpretation of the findings or the magnitude of the estimates (< 1% change).

## Take-Home Points

### Research Question:

How does metabolic bariatric surgery affect OSA endotypes (loop gain, arousal threshold, muscle compensation), and do baseline endotypes predict improvements in OSA severity?

### Results:

Metabolic bariatric surgery was associated with improvements in apnea-hypopnea index, BMI, upper airway collapsibility, and loop gain as well as reductions in arousal threshold. Those with less-collapsible upper airways and low loop gain at baseline were more likely to see improvements in their OSA severity after surgery.

### Interpretation:

Our results indicate that weight loss via metabolic bariatric surgery improves key physiological traits underlying OSA and may be particularly effective in patients with less-collapsible airways and low loop gain at baseline.

## ABBREVIATIONS:

AHI: apnea-hypopnea index
GLME: generalized linear mixed effects
MBS: metabolic bariatric surgery
NREM: non-rapid eye movement
OAGB: one-anastomosis gastric bypass
PSG: polysomnogram
SG: sleeve gastrectomy
