# Supplementary material for: The Impact of Weight Loss on the Physiological Endotypes Associated With OSA
Source: Chest. Author manuscript; Available in PMC 2026 Jul 21. (PMC13384779; doi:10.1016/j.chest.2026.02.003)
Supplement: sup [file NIHMS2150625-supplement-sup.docx]

**SUPPLEMENTAL MATERIAL**

**The impact of weight loss on the physiological endotypes associated with OSA**

**Supplementary Methods**

*Rationale for combining multiple datasets:*

Originally, we designed and commenced a prospective trial (BAROSA trial) to assess our research questions. However, due to the COVID-19 pandemic and lockdowns in Melbourne Australia, elective surgeries were delayed or cancelled during the trial period, and we were not permitted to study participants in the laboratory. Consequently, the recruitment targets for our prospective trial were not met within the timeframe set for the study (dictated by funding). As such, consent was obtained from the Monash University Human Research Ethics Committee (Project ID’s: 20346 and 14937) to combine our data with data from two studies Bakker et al.^1^ and Sutherland et al.^2^ to answer our research questions. A summary of the methods utilised in each of trials included in our analysis are detailed below.

1. *BAROSA Trial*

The BAROSA Trial was designed to assess the impacts of weight loss via metabolic bariatric surgery (MBS) on the obstructive sleep apnea (OSA) endotypes. Inclusion criteria were as follows; aged between 18-65 years, had an apnea-hypopnea index (AHI) >15 events/hr and a body mass index (BMI) between 35kg/m^2^ to 55kg/m^2^. Participants treated with continuous positive airway pressure (CPAP) were required to abstain from CPAP use for 5 days before assessment and were otherwise healthy (except for treated hypertension and diabetes). Participants were excluded if they had any untreated medical conditions, took medication affecting ventilation or muscle control, had previous surgery for OSA or obesity, had obesity hypoventilation syndrome, were pregnant or breastfeeding or had a recent history of drowsy driving. The study was approved by the Alfred Health Ethics Committee (Project ID:6225) and registered with Monash University Ethics Committee (Project ID:28299). The protocol was registered on the Australian and New Zealand Clinical Trials Registry (ACTRN12619001505190). All participants provided their written and informed consent to participate. Data were managed using REDCap electronic data capture tools hosted at Monash University^3,4^.

To measure weight loss’s impact on OSA severity and OSA endotypes, participants underwent four overnight polysomnograms (PSG), two before MBS (sleeve gastrectomy [SG]) and two within 6-12 months after surgery. One of these PSGs involved a standard clinical montage to assess OSA severity (and the OSA endotypes using the PSG method) and the other PSG was a research PSG (detailed in the main document) that aimed to assess the OSA endotypes using gold standard techniques. At these visits, height and weight were measured with a wall-mounted stadiometer and calibrated digital scale. Overnight monitoring included electroencephalogram, chin electromyogram, electrooculogram, electrocardiogram, respiratory effort, oxygen saturation, snoring, body position and airflow (for the clinical PSG) using a nasal cannula and oronasal thermistor. Sleep state, arousal and respiratory events were scored using AASM criteria^5^. Specifically, hypopneas were scored if the airflow signal was reduced by ≥30% of baseline with ≥3% oxygen desaturation or cortical arousal. A total of 12 participants completed the trial and were included in the analysis.

1. *ABC Trial*

The “Apnea, Bariatric Surgery Versus Continuous Positive Airway Pressure (CPAP) Trial (ABC)” by Bakker et al was a randomized control trial to compare the management of OSA with CPAP versus MBS^1^. Participants who were randomized to receive surgery had laparoscopic gastric banding (LAGB). Participants underwent an overnight attended PSG in the laboratory at baseline and then at 9 and 18 months post-surgery. For this study, only the 18-month follow-up data was used in the analysis. The 18 month data was used in this analysis because it represented the most complete data set and it gave the longest follow up period to observe maximal weight loss. The inclusion criteria were age between 18-65 years, a BMI 35–45 kg/m^2^, severe OSA (AHI > 30 events/h [level 1study] or AHI > 20 events/h [level 3 study]), and at least one OSA symptom. Participants who had prior use of CPAP within the last two years or previous MBS were excluded. Those who had hypoventilation syndrome, increased perioperative risk, drowsy driving in the past year, were not fluent in English, or had any unstable medical conditions were also excluded. The study was approved by the institutional review boards at Brigham and Women’s Hospital and Beth Israel Deaconess Medical Center. The protocol was registered at clinicaltrials.gov (NCT01187771). All participants provided their written and informed consent to participate in the study.

Participants completed attended polysomnography using the Compumedics E-Series system (Abbotsford, Victoria Australia).  The PSG montage included three cortical electroencephalograms, bilateral electro-oculograms, chin electromyogram, thoracic and abdominal respiratory inductance plethysmography, airflow measured via oronasal thermistor and nasal cannula, electrocardiography, bilateral tibialis electromyograms, and finger pulse oximetry. Apneas were scored as a >90% reduction in breathing amplitude for ≥10 seconds; hypopneas were scored as >30% reduction in breathing amplitude associated with SpO_2_ desaturation of ≥3% for ≥10 seconds.  The AHI was calculated as the number of apneas plus hypopneas divided by total sleep time.

Of the 49 randomized participants, 28 were randomized to the surgery group and 21 to the CPAP group. Of the 28 in the surgery group, 14 had LAGB, 10 had CPAP, and 4 had neither. In the CPAP group, 20 had CPAP, and 1 had CPAP and then had gastric bypass surgery outside of the RCT. For this analysis, the 14 participants in the surgery group and 1 participant in the CPAP group who had surgery were included. In addition, the data for a participant who had their surgery delayed and was not included in the original trial was also included. Due to the delay in surgery, their 9-month follow-up data was used. A total of 16 participants were included in the analysis from this trial.

1. *Sutherland Trial*

The aim of this trial was to establish the effect of weight loss from MBS on the upper airway volume and soft tissues^2^. Participants underwent either a SG or a One Anastomosis Gastric Bypass (OAGB). Participants underwent an overnight attended laboratory PSG in the clinical sleep laboratory of The Royal North Shore Hospital before and 6 months after surgery. Participants undergoing surgery were recruited from a private clinic in Sydney, Australia. Inclusion criteria were OSA at baseline with an AHI ≥ 5 events/hour. Participants were excluded if they had a known history of syndromal craniofacial abnormalities, previous craniofacial or upper airway surgery, or significant upper airway deformity or obstruction that was not obesity related (e.g. enlarged tonsils, or nasal obstruction); contraindications to magnetic resonance imaging (MRI) (incompatible implants, claustrophobia, pregnancy, exceeding scanner size limits [weight > 200 kg, waist circumference > 220 cm]); or dental work causing artefact. The study was approved by the Northern Sydney Local Health District Human Research Ethics Committee (HREC/15/HAWKE/386, RESP/15/278). All participants gave written informed consent.

A standard PSG (Compumedics, Melbourne, Australia) was performed with channels for electroencephalogram, electrooculogram, chin electromyogram, electrocardiogram, anterior tibial electromyogram, nasal pressure, chest and abdomen movements, body position and arterial oxygen saturation (SpO_2_). All studies were scored in accordance with the 2012 American Academy of Sleep Medicine (AASM) scoring manual by experienced scorers. Specifically, hypopneas were scored if the airflow signal was reduced by ≥30% of baseline with ≥3% oxygen desaturation or cortical arousal.

**Supplemental Results**

***SECTION 1: Between Trial Comparisons***

A breakdown of the participants baseline characteristics and change in characteristics by trial can be found in e-Table 1. There was a difference in baseline age, BMI, AHI and upper airway collapsibility. There was also a significant difference in weight change and BMI change between the three trials. The difference in weight and BMI change between the trials may reflect the different types of surgery in each study and the variations in the post-surgery follow up period 6-18 months (max weight loss is typically at 6 months). Importantly, there was no difference in change of any of the OSA endotypes between trials.

***e-Table 1: Comparisons Between Baseline and Change Characteristics Between Trials***

| Characteristic | Group 1  Barosa Trial  N = 12*^1^* | Group 2  ABC Trial  N = 16*^1^* | Group 3  Sutherland et al., 2023  N = 15*^1^* | p-value*^2^* |
| --- | --- | --- | --- | --- |
| Baseline characteristic |  |  |  |  |
| Age (years) | 41.3 ± 11.1 | 51.7 ± 8.8^a^ | 47.2 ± 8.1 | 0.019 |
| Weight (kg) | 135.0 ± 23.5 | 115.5 ± 20.3 | 120.9 ± 20.7 | 0.062 |
| BMI (kg/m^2^) | 45.5 ± 5.6 | 39.4 ± 2.9^a^ | 42.4 ± 6.2 | 0.010 |
| AHI (events/hr) | 60.3 ± 32.1 | 58.7 ± 26.4^b^ | 34.6 ± 12.9 | 0.004 |
| Upper Airway Collapsibility (% Veupnea) | 66.1 [36.5, 77.3]^b^ | 69.3 [65.1, 73.5]^b^ | 78.4 [75.7, 82.5] | 0.003 |
| Loop Gain | 0.7 [0.5, 0.8] | 0.6 [0.6, 0.7] | 0.6 [0.5, 0.7] | 0.669 |
| Arousal Threshold (% Veupnea) | 156.1 ± 36.2 | 157.3 ± 29.4 | 136.6 ± 14.9 | 0.087 |
| Muscle Compensation (% Veupnea) | 2.1 [-0.4, 6.2] | 3.8 [-9.1, 12.2] | 4.5 [2.4, 8.0] | 0.661 |
|  |  |  |  |  |
| Change in Characteristic |  |  |  |  |
| ΔWeight (kg) | -33.4 ± 11.6 | -16.2 ± 8.8^a^ | -33.5 ± 7.2 | <0.001 |
| ΔBMI (kg/m^2^) | -11.2 ± 3.4 | -5.6 ± 3.1^ab^ | -11.8 ± 2.3 | <0.001 |
| ΔAHI (events/hr) | -33.4 ± 20.7 | -25.5 ± 20.9 | -21.1 ± 16.3 | 0.265 |
| ΔUpper Airway Collapsibility (% Veupnea) | 6.1 [-2.5, 13.9] | 4.0 [-2.6, 9.5] | -0.7 [-4.3, 5.4] | 0.330 |
| ΔLoop Gain | -0.1 ± 0.2 | 0.0 ± 0.1 | -0.1 ± 0.2 | 0.153 |
| ΔArousal Threshold (% Veupnea) | -7.2 ± 12.7 | -1.3 ± 20.4 | -13.1 ± 15.5 | 0.166 |
| ΔMuscle Compensation (% Veupnea) | -1.6 [-10.0, 6.1] | -6.9 [-12.2, 0.3] | 0.0 [-2.6, 3.0] | 0.252 |
| BMI = body mass index, AHI = apnoea hypopnea index *^1^*Median [Q1, Q3]; Mean ± SD *^2^*One-Way Analysis of Variance, Welch's One-Way Analysis of Variance, Kruskal-Wallis’s rank sum test  ^a^Post hoc test indicated a significant difference compared to group 1  ^b^ Post hoc test indicated a significant difference compared to group 3 | | | |  |

***SECTION 2: Between Sex Comparisons***

A breakdown of the participants baseline characteristics and change in characteristics by sex can be found in e-Table 2. There was a difference in baseline weight, upper airway collapsibility and loop gain between the sexes. However, MBS impacted the change in OSA severity and endotypes by similar amounts between the sexes.

**e-Table 2: Comparisons Between Baseline and Change Characteristics by Sex**

| Characteristic | Male N = 18^1^ | Female N = 25^1^ | Difference estimate  [95% CI] | p-value |
| --- | --- | --- | --- | --- |
| Baseline Characteristic |  |  |  |  |
| Age (years) | 45.0 ± 10.6 | 48.8 ± 9.4 | -3.5 [-9.6 to 2.7] | 0.260 |
| BMI (kg/m^2^) | 42.4 ± 5.0 | 41.9 ± 5.9 | 0.2 [-3.2 to 3.6] | 0.905 |
| Weight (kg) | 132.0 [126.8, 144.2] | 108.3 [98.8, 123.1] | 23.3 [11.2 to 35.4] | 0.0003 |
| AHI (events/hr) | 61.8 ± 32.3 | 42.7 ± 18.9 | '9.1 [-6.4 to 24.6] | 0.245 |
| Upper Airway Collapsibility (%V_eupnea_) | 68.1 [36.8, 76.1] | 76.0 [69.7, 81.9] | -4.5 [-16.2 to 7.2] | 0.440 |
| Loop Gain | 0.68 [0.61, 0.72] | 0.57 [0.44, 0.72] | 0.06 [-0.05 to 0.18] | 0.266 |
| Arousal Threshold (%V_eupnea_) | 160.4 ± 34.5 | 142.0 ± 21.1 | 10.6 [-6.5 to 27.7] | 0.217 |
| Muscle Compensation (%V_eupnea_) | 3.4 [-4.0, 11.1] | 4.2 [0.7, 7.1] | -4.1 [-12.6 to 4.4] | 0.337 |
|  |  |  |  |  |
| Change in Characteristic |  |  |  |  |
| ΔBMI (kg/m^2^) | -8.4 ± 4.4 | -10.0 ± 3.8 | 1.1 [-1.0 to 3.2] | 0.280 |
| ΔWeight (kg) | -26.8 ± 13.8 | -27.2 ± 11.4 | -1.1 [-7.5 to 5.2] | 0.721 |
| ΔAHI (events/hr) | -18.1 [-41.0, -12.4] | -24.7 [-35.0, -12.1] | 1.1 [-10.9 to 13.1] | 0.856 |
| ΔUpper Airway Collapsibility (%V_eupnea_) | -0.9 [-3.3, 10.3] | 3.0 [-2.9, 8.2] | 1.6 [-7.8 to 10.9] | 0.738 |
| ΔLoop Gain | -0.07 ± 0.18 | -0.06 ± 0.14 | -0.01 [-0.11 to 0.09] | 0.878 |
| ΔArousal Threshold (%V_eupnea_) | -3.4 ± 19.4 | -9.8 ± 15.3 | 6.4 [-4.0 to 16. 9] | 0.223 |
| ΔMuscle Compensation (%V_eupnea_) | -3.2 [-14.0, 5.0] | -0.6 [-7.5, 2.6] | 0.6 [-10.7 to 12.0] | 0.912 |

_1_Mean ± SD; n (%); Median [Interquartile Range] depending on whether the model residuals were normally distributed. Generalised linear mixed-effects model of the order: Baseline Characteristic ~ Sex + (1| Study Source) + (1| ID). BMI = Body mass index, AHI = Apnea-hypopnea index

***SECTION 3: OSA Endotypes Derived from the Research PSG***

A total of 12 people participated in the original study. However, only 10 participants had all four obstructive sleep apnea (OSA) endotypes obtained before and after surgery via research PSG for analysis due to COVID restrictions during data collection. Therefore, only data from these 10 participants are reported here. Most participants were male (70%), were close to middle age (mean age was 40.2 years), were obese (mean body mass index [BMI] = 46.0 kg/m^2^) and had very severe OSA (apnea-hypopnea index [AHI] = 67.7 events/hr). All participants underwent SG. See e-Table 3 for further details on baseline participant characteristics in this subset.

***e-Table 3: Baseline Participant Characteristics***

|  | N = 10*^1^* |
| --- | --- |
| Age (years) | 40.2 ±12.0 |
| Gender, male | 7 (70%) |
| Weight (kg) | 137.6 ± 25.0 |
| BMI (kg/m^2^) | 46.0 ± 6.0 |
| AHI (events/hr) | 67.7 ± 34.6 |
| Surgery Type |  |
| Sleeve Gastrectomy (SG) | 10 (100%) |
| BMI = body mass index, AHI = Apnea Hypopnea Index *^1^*Mean ± SD; n (%); Median [Interquartile Range] | |

OSA severity and endotypes (derived from the Research PSG) before and after MBS are presented in e-Table 4 and e-Figure 1.

**e-Table 4: Impact of Metabolic Bariatric Surgery on OSA Severity and Endotypes derived from the Research PSG**

|  | Baseline | Follow Up | Difference Estimate [95% CI] | p-value |
| --- | --- | --- | --- | --- |
| AHI (events/hr) | 67.7 ± 34.6 | 33.4 ± 30.3 | -34.3 [-48.7 to -20.0] | <0.001 |
| AHI NREM (events/hr) | 67.1 ± 36.2 | 33.2 ± 31.8 | -33.8 [-50.0 to -17.7] | <0.001 |
| Veupnea (L/min) | 8.1 ± 1.2 | 7.1 ± 0.9 | -1.1 [-1.9 to -0.3] | 0.011 |
| Upper Airway Collapsibility | |  |  |  |
| Vpassive (L/min) | -0.7 ± 5.1 | 3.2 ± 3.3 | 3.9 [2.4 to 5.3] | <0.001 |
| Vactive (L/min) | 0.9 ± 6.2 | 4.7 ± 3.1 | 3.8 [1.5 to 6.1] | 0.002 |
| Loop Gain | 3.9 ± 1.3 | 2.3 ± 1.1 | -1.5 [-2.6 to -0.5] | 0.008 |
| Arousal Threshold (L/min) | 12.7 ± 3.7 | 9.8 ± 2.9 | -2.9 [-5.6 to -0.1] | 0.041 |
| Upper Airway Gain (L/min) | 0.3 [0.0, 0.5] | 0.4 [0.1, 0.6] | -0.6 [-1.7 to 0.5] | 0.277 |
| Data shown in Baseline and Follow-up columns represent Mean ± SD or Median (Q1-Q3). Difference estimate generated using generalised linear mixed-effects model of the order: AHI/Endotype ~ Visit + (1\|Study Source) + (1\|Participant). AHI = Apnea-hypopnea index, NREM = non-rapid eye movement sleep. | | | | |


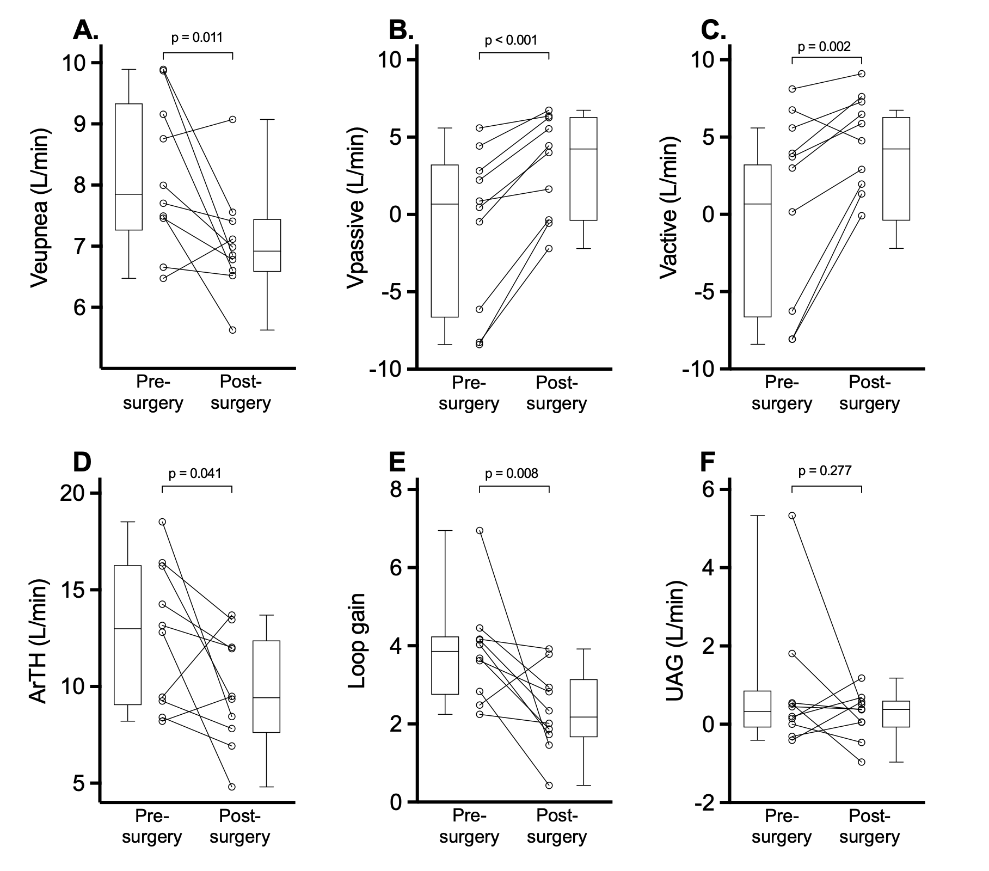
Following MBS, participants had a significant improvement in OSA severity (total AHI reduced from 67.7 ± 34.6 to 33.4 ± 30.3 events/hr). There was a significant improvement in upper airway collapsibility (Vpassive and Vactive) as well as a reduction in the steady-state loop gain and arousal threshold following weight loss. There was no significant change in muscle compensation (upper airway gain) following weight loss.

e-Figure 1: Endotypes before and after MBS as measured during the research PSG**.** Following weight loss surgery, there were significant changes in resting ventilation during sleep (Veupnea, panel A), both passive and active upper airway collapsibility (Vpassive and Vactive respectively, panels B and C), loop gain (panel D) and the arousal threshold (ArTH, panel E); however, there was no significant change muscle compensation (i.e. upper airway gain [UAG], panel F).

***SECTION 4: Relationship between the duration of follow-up and the changes OSA severity, body weight and the OSA endotypes.***

In order to assess the association between the duration of follow-up after metabolic bariatric surgery and the magnitude of change in OSA severity and underlying pathophysiological traits, we performed generalised linear mixed-effects models with follow-up duration (in months) as a fixed effect and random intercepts for study source and participant (see e-Table 5).

***e-Table 5: Per month change in key outcome variables***

| **Variable** | **Estimate** | **95% Confidence intervals** | **p-value** |
| --- | --- | --- | --- |
| AHI Total (events/hr) | -1.88 | -2.41 to -1.35 | <0.001 |
| Weight (kg) | -1.62 | -2.12 to -1.12 | <0.001 |
| BMI (kg/m^2^) | -0.54 | -0.71 to -0.37 | <0.001 |
|  |  |  |  |
| Upper Airway Collapsibility (%V_eupnea_) | 0.62 | 0.12 to 1.06 | 0.005 |
| Loop Gain | -0.003 | -0.71 to -0.37 | 0.101 |
| Arousal Threshold (%V_eupnea_) | -0.37 | -0.71 to -0.37 | 0.093 |
| Muscle Compensation (%V_eupnea_) | -0.26 | -0.71 to -0.37 | 0.210 |
| Difference estimate (unstandardised beta and 95% CI) using generalised linear mixed-effects model of the order: Variable ~ Follow-up duration + (1\|Study Source) + (1\|Participant). | | | |

Over the follow-up period, total AHI decreased by 1.88 events/h per month, with corresponding reductions in weight (−1.62 kg/month) and BMI (−0.54 kg/m² per month). Among the OSA endotypes, upper airway collapsibility improved significantly (+0.62 %V_eupnea_ per month;), whereas loop gain, arousal threshold, and muscle compensation showed no significant longitudinal change. The absence of a significant linear trend for loop gain and arousal threshold may reflect non-linear trajectories, with the majority of improvement occurring early (within the first 6–12 months) and plateauing thereafter, consistent with the significant pre-to-post reductions observed in main analyses.

***SECTION 5: Dichotomising Responders***

In addition to the main analyses, participants were categorized as responders or non-responders based on three distinct criteria: 1) if their AHI was reduced by ≥ 50% of baseline, 2) if their AHI was reduced by ≥ 50% of baseline with a follow-up AHI was ≤15 events/hr and 3) if their follow-up AHI was ≤15 events/hr (i.e. those that had no OSA or mild OSA following MBS).

In total, 22 (51.2%) participants had a 50% reduction in AHI following surgery (responder 1 criteria), 15 (34.9%) participants had a 50% reduction in AHI with a follow-up AHI ≤ 15 events/hr (responder 2 criteria) and 20 (46.5%) had a follow-up AHI ≤ 15 events/hr (responder 3 criteria). When using criteria 1, the baseline OSA endotypes were not different between responders vs non-responders. However when using criteria 2 or 3, responders tended to have milder collapsibility and a lower loop gain compared to non-responders. These results are detailed below in e-Table 6.

|  | **Responder 1 Criteria:**  ≥ 0% reduction in AHI | | | | **Responder 2 Criteria:**  >50% reduction in AHI & <15 events/hr post-MBS | | | | **Responder 3 Criteria:**  <15 events/hr post-MBS | | | | |
| --- | --- | --- | --- | --- | --- | --- | --- | --- | --- | --- | --- | --- | --- |
| Characteristic | Responder,  N = 22*^1^* | Non-Responder,  N = 21*^1^* | Difference estimate  [95% CI] | p-value | Responder,  N = 15*^1^* | Non-Responder,  N = 28*^1^* | Difference estimate  [95% CI] | p-value | Responder,  N = 20*^1^* | Non-Responder,  N = 23*^1^* | Difference estimate  [95% CI] | p-value |  |
| Baseline Characteristics | | | | | | | | | | | | | |
| Age (years) | 44.9 ± 10.1 | 49.6 ± 9.5 | 3.9 [-9.6 to 1.8] | 0.172 | 42.9 ± 10.0 | 49.6 ± 9.3 | -6.1 [-12.1 to -0.1] | 0.045 | 45.9 ± 10.3 | 48.4 ± 9.8 | -1.6 [-7.9 to 4.6] | 0.604 |  |
| Gender, male | 6 (27.3%) | 12 (57.1%) | -1.6 [-3.1 to -0.04] | 0.045 | 3 (20.0%) | 15 (53.6%) | -1.4 [-3.1 to -0.2] | 0.089 | 4 (20.0%) | 14 (60.9%) | -1.7 [-3.2 to -0.1] | 0.033 |  |
| BMI (kg/m^2^) | 42.0 ± 4.8 | 42.3 ± 6.2 | -1.0 [-4.2 to 2.1] | 0.512 | 42.6 ± 5.0 | 41.9 ± 5.7 | -0.2 [-3.6 to 3.3] | 0.915 | 42.2 ± 4.9 | 42.1 ± 6.0 | -1.1 [-4.5 to 2.3] | 0.523 |  |
| Weight (kg) | 118.9 ± 20.0 | 126.9 ± 24.3 | -10.1 [-22.9 to 2.7] | 0.120 | 122.2 ± 20.0 | 123.2 ± 23.8 | -2.7 [-17.1 to 11.7] | 0.707 | 118.8 ± 19.9 | 126.3 ± 24.2 | -10.6 [-24.5 to 3.3] | 0.130 |  |
| AHI Total (events/hr) | 50.8 ± 23.2 | 50.6 ± 30.8 | 3.1 [-11.0 to 17.1] | 0.663 | 41.9 ± 17.9 | 55.4 ± 29.8 | -6.1 [-21.5 to 9.3] | 0.427 | 37.4 ± 17.4 | 62.3 ± 28.5 | -18.3 [-33.0 to -3.7] | 0.016 |  |
| Upper Airway Collapsibility  (%V_eupnea_) | 75.1 [66.2, 78.5] | 72.1 [63.0, 80.4] | .5 [-3.8 to 16.8] | 0.211 | 78.1 [68.2, 81.0] | 71.9 [62.0, 77.3] | 7.4 [-3.9 to 18.7] | 0.194 | 78.2 [70.8, 82.2] | 70.2 [42.6, 74.9] | 12.4 [1.3 to 23.4] | 0.029 |  |
| Loop Gain | 0.57 [0.44, 0.68] | 0.65 [0.60, 0.78] | -0.10 [-0.20 to 0.00] | 0.055 | 0.47 [0.43, 0.65] | 0.66 [0.60, 0.74] | -0.11 [-0.22 to -0.00] | 0.048 | 0.57 ± 0.17 | 0.71 ± 0.18 | -0.13 [-0.24 to -0.02] | 0.019 |  |
| Arousal Threshold (%V_eupnea_) | 144.1 ± 20.4 | 155.6 ± 34.8 | -8.8 [-24.2 to 6.7] | 0.259 | 139.1 ± 17.0 | 155.4 ± 32.1 | -10.0 [-26.9 to 7.0] | 0.242 | 138.0 ± 16.0 | 159.9 ± 33.3 | -15.8 [-32.5 to 0.9] | 0.062 |  |
| Muscle Compensation (%V_eupnea_) | 4.2 [0.2, 12.5] | 3.7 [-4.4, 6.5] | 5.2 [-3.2 to 13.5] | 0.218 | 3.4 [0.4, 8.9] | 4.0 [-3.6, 8.2] | 2.8 [-6.1 to 11.6] | 0.530 | 4.1 [0.5, 6.6] | 3.8 [-3.8, 11.0] | 1.8 [-6.7 to 10.2] | 0.677 |  |
|  |  |  |  |  |  |  |  |  |  |  |  |  |  |
| Change in Characteristic | | | | | | | | | | | | | |
| ΔBMI Change (kg/m^2^) | -10.3 ± 3.3 | -8.3 ± 4.7 | -1.1 [-2.9 to 0.8] | 0.251 | -11.9 ± 2.3 | -8.0 ± 4.2 | -2.3 [-4.3 to -0.4] | 0.022 | -11.4 ± 2.8 | -7.5 ± 4.3 | -2.1 [-4.1 to -0.1] | 0.038 |  |
| ΔWeight Change (kg) | -29.3 ± 10.5 | -24.6 ± 13.8 | -2.1 [-7.8 to 3.6] | 0.467 | -34.1 ± 8.2 | -23.3 ± 12.6 | -6.4 [-12.4 to -0.4] | 0.036 | -32.1 ± 9.2 | -22.6 ± 13.1 | -4.6 [-10.8 to 1.5] | 0.137 |  |
| ΔAHI Change (events/hr) | -33.5 [-42.3, -23.0] | -12.4 [-19.5, -9.3] | -22.2 [-31.9 to -12.5] | <0.001 | -28.5 [-38.0, -20.7] | -15.5 [-35.1, -10.3] | -11.0 [-23.2 to 1.1] | 0.073 | -22.7 [-34.3, -12.1] | -19.8 [-35.9, -12.4] | -2.3 [-14.1 to 9.6] | 0.703 |  |
| ΔAHI Change (%) | -71.5 [-76.5, -63.7] | -29.8 [-45.9, -20.5] | -45.2 [-56.1 to -34.2] | <0.001 | -73.8 [-83.2, -67.3] | -42.7 [-53.0, -25.4] | -39.6 [-53.6 to -25.6] | <0.001 | -71.5 [-79.9, -55.2] | -32.6 [-60.6, -22.4] | -33.3 [-47.8 to -18.8] | <0.001 |  |
| ΔUpper Airway Collapsibility  (%V_eupnea_ ) | 6.5 [1.9, 9.9] | -2.1 [-4.7, 5.4] | 3.6 [-5.6 to 12.7] | 0.434 | 3.0 [-0.8, 9.1] | -0.7 [-3.4, 10.1] | -1.3 [-10.9 to 8.3] | 0.785 | 0.6 [-3.9, 8.5] | 5.2 [-2.6, 10.6] | -6.2 [-15.2 to 2.8] | 0.171 |  |
| ΔLoop Gain Change | -0.08 ± 0.17 | -0.05 ± 0.15 | -0.02 [-0.12 to 0.07] | 0.598 | -0.08 ± 0.19 | -0.06 ± 0.13 | -0.02 [-0.12 to 0.08] | 0.756 | -0.08 ± 0.18 | -0.05 ± 0.14 | -0.02 [-0.12 to 0.07] | 0.609 |  |
| ΔArousal Threshold (%V_eupnea_) | -13.9 ± 13.5 | 0.0 ± 18.1 | -13.9 [-23.4 to -4.3] | 0.006 | -15.6 ± 14.6 | -2.5 ± 17.0 | -13.0 [-23.3 to -2.8]' | 0.014 | -12.1 ± 14.5 | -2.7 ± 18.5 | 9.4 [-19.5 to 0.7] | 0.068 |  |
| ΔMuscle Compensation  (%V_eupnea_) | -0.3 [-6.4, 3.3] | -2.6 [-14.5, 0.5] | 6.1 [-4.5 to 16.7] | 0.253 | -0.0 [-6.3, 3.2] | -2.5 [-11.4, 1.4] | 6.5 [-4.7 to 17.8] | 0.249 | -0.3 [-6.1, 3.2] | -4.0 [-15.3, 1.4] | 7.2 [-3.5 to 17.9] | 0.180 |  |
| BMI = body mass index, AHI = apnea hypopnea index, SD = standard deviation  *^1^*Mean ± SD; n (%); Median [Interquartile Range] depending on whether the model residuals were normally distributed  Generalised linear mixed-effects model of the order: Characteristic ~ Responder + (1\| Study Source) + (1\| ID)) | | | | | | | | | | | | | |

***e-Table 6: Differences in baseline (and change in) characteristics between responders and non-responders***

Receiver operating characteristic (ROC) analyses were performed to evaluate the ability of several baseline physiological variables to discriminate between responders and non-responders to surgery. e-Table 7 summarises the area under the curve (AUC), optimal classification thresholds (using Youden’s J criterion), and associated diagnostic performance indices for each predictor.

***e-Table 7: ROC results for all 3 Responder Criteria***

| **Predictor** | **AUC** | **Threshold** | **Sensitivity** | **Specificity** | **False Positive Rate** | **Positive Predictive Value** | **Negative Predictive Value** |
| --- | --- | --- | --- | --- | --- | --- | --- |
| *Responder Criteria #1* | | | | | | | |
| Upper Airway Collapsibility  (%V_eupnea_) | 0.54 | 76.1 | 0.50 | 0.71 | 0.29 | 0.66 | 0.58 |
| Loop Gain | 0.68 | 0.58 | 0.55 | 0.81 | 0.19 | 0.75 | 0.63 |
| Arousal Threshold (%V_eupnea_) | 0.57 | 185.2 | 1.00 | 0.29 | 0.71 | 0.59 | 1.00 |
| AHI Total (events/hr) | 0.56 | 32.5 | 0.82 | 0.48 | 0.53 | 0.62 | 0.71 |
|  |  |  |  |  |  |  |  |
| *Responder Criteria #2* | | | | | | | |
| Upper Airway Collapsibility  (%V_eupnea_) | 0.64 | 76.1 | 0.67 | 0.75 | 0.25 | 0.59 | 0.81 |
| Loop Gain | 0.72 | 0.46 | 0.53 | 0.93 | 0.07 | 0.80 | 0.79 |
| Arousal Threshold (%V_eupnea_) | 0.63 | 157.8 | 0.93 | 0.39 | 0.61 | 0.45 | 0.92 |
| AHI Total (events/hr) | 0.60 | 51.6 | 0.8 | 0.54 | 0.46 | 0.48 | 0.83 |
|  |  |  |  |  |  |  |  |
| *Responder Criteria #3* | | | | | | | |
| Upper Airway Collapsibility  (%V_eupnea_) | 0.75 | 76.0 | 0.70 | 0.83 | 0.17 | 0.78 | 0.76 |
| Loop Gain | 0.73 | 0.58 | 0.60 | 0.83 | 0.17 | 0.75 | 0.70 |
| Arousal Threshold (%V_eupnea_) | 0.70 | 157.8 | 0.95 | 0.48 | 0.52 | 0.61 | 0.92 |
| AHI Total (events/hr) | 0.76 | 51.6 | 0.85 | 0.65 | 0.35 | 0.68 | 0.83 |

The predictive performance of baseline physiological traits varied markedly across the three responder definitions. Using the Youden’s J criterion to optimise thresholds, discrimination was modest under Responder Criteria #1 (AUC 0.54–0.68), with Loop Gain showing the highest accuracy (AUC 0.68, sensitivity 0.55, specificity 0.81). Predictive performance improved under Responder Criteria #2 (AUC 0.60–0.72), with Loop Gain again the strongest classifier (AUC 0.72, sensitivity 0.53, specificity 0.93, NPV 0.79) and collapsibility also performing well (AUC 0.64). The strongest discrimination occurred with Responder Criteria #3 (AUC 0.70–0.76), where baseline AHI (AUC 0.76, sensitivity 0.85, specificity 0.65) and collapsibility (AUC 0.75, sensitivity 0.70, specificity 0.83) provided the best combination of sensitivity and specificity. Overall, physiological traits exhibited only modest predictive utility under lenient responder definitions but demonstrated substantially stronger discrimination when stricter clinical response thresholds were applied.

***SECTION 6: How does the change in the Endotypes relate to the Minimal Detectable Differences versus are they Clinically Meaningful?***

Although statistically significant, the observed improvements in collapsibility (V_passive_ 8.7–11.8%V_eupnea_) and loop gain (–0.07) were smaller than the individual-level minimum detectable differences (MDDs) reported previously^10,11^ (MDDs for Collapsibility=22-23%V_eupnea_; Loop gain=0.17–0.20). However, it is worth noting that these MDDs were derived from consecutive-night studies subject to first-night and carry-over effects, likely overestimating measurement error, and were calculated for individual rather than group comparisons (where detectable thresholds scale down by ~√N). Furthermore, MDD is a statistical measure of reliability rather than an indicator of a clinically meaningful difference. A clear example is the AHI: Strassberger et al.^10^ reported an MDD of ~27 events/h, yet both CPAP (≈ –23 events/h) and oral appliances (≈ –14 events/h) are considered clinically effective by AASM guidelines despite falling below this threshold^12,13^.

Accordingly, we attempted to interpret our observed endotype changes against published intervention anchors rather than individual‑level MDDs. Specifically, when we benchmarked the change in collapsibility (per V_passive_) and loop gain against interventions targeting the same traits (Collapsibility: mandibular advancement devices^14^, upper airway surgery^7^, body position^15^, Loop gain: acetazolamide^16^, oxygen therapy^14^, and suthiame^18^), weight-loss effects on V_passive_ and loop gain were smaller in magnitude yet the reductions in AHIs across studies were relatively similar (see e-Table 8).

***e-Table 8: Comparison of change in OSA endotypes and OSA severity with interventions targeting collapsibility and loop gain***

| **Study** | **Intervention** | **Endotype altered** | **Change in Endotype**  **Mean difference (SD)** | **% Reduction in AHI** |
| --- | --- | --- | --- | --- |
| Current study | Weight loss | Collapsibility (%V_eupnea_) | 8.7 ± 19.1% | 51 |
|  |  | Loop Gain | -0.07 ± 0.15 |  |
|  |  |  |  |  |
| Wang et al. 2024^15^ | Lateral positioning (non-positional OSA) | Collapsibility (%V_eupnea_) | 7.3 ± 21.2% | 16 |
|  | Lateral positioning (supine predominant OSA) | Collapsibility (%V_eupnea_) | 12.3 ± 21.3% | 79 |
|  |  |  |  |  |
| Wong et al. 2022^7^ | Surgery | Collapsibility (%V_eupnea_) | 19.4 ± 39.5% | 43 |
|  |  |  |  |  |
| Edwards et al. 2025^14^ | MAD | Collapsibility (%V_eupnea_) | 22.4 ± 31.2% | 56 |
|  | Oxygen | Loop Gain | -0.15 ± 0.16 |  |
|  |  |  |  |  |
| Sands et al. 2024^16^ | Acetazolamide | Loop Gain | -0.13 ± 0.17 | 34 |
|  |  |  |  |  |
| Hedner et al. 2022/ Hoff et al. 2024^18,19^ | Sulthiame (200mg) | Loop Gain | -0.13 ± 0.11 | 34 |
|  | Sulthiame (400mg) | Loop Gain | -0.17 ± 0.09 | 40 |

MAD: Mandibular advancement devices. SD: standard deviation of the mean difference. A negative value for the change in loop gain denotes a reduction. A positive value for the change in collapsibility reflects an improvement in airway collapsibility (i.e. less collapsible).

These smaller changes observed in our study may partly reflect methodological factors. Body position was not controlled in the primary analysis, and V_passive_ improvements were notably larger (~12%) in the reduced subset measured during NREM supine sleep. Moreover, the comparator studies typically enrolled individuals with substantially worse baseline collapsibility, making larger absolute changes more achievable. Changes in loop gain may also reflect a secondary consequence of AHI reduction rather than a direct physiological effect of weight loss.

Based on published intervention studies, changes of the magnitude observed here in the endotypes would be expected to yield only modest AHI improvements if they occurred in isolation. However, while individually modest, the simultaneous multi‑trait shifts we observed (i.e., concurrent improvements in V_passive_ and loop gain) plausibly account for the overall reduction in AHI. This aligns with the multifactorial nature of OSA pathophysiology, wherein comparable improvements in AHI can result either from a large change in a single trait or from coordinated, smaller changes across multiple traits. Future work is needed to establish OSA‑specific minimal clinically important differences using patient‑anchored outcomes to more precisely contextualize these findings.

***SECTION 7: Is the Reduction in Loop Gain Driven by Weight-loss? Comparison of Reductions in Loop Gain Following OSA Treatment***

While the current study found that MBS decreased loop gain, there are several reasons to indicate that the reduction may not be a direct cause of weight loss but rather the result of a reduction in OSA severity. If weight loss did have a causal effect on loop gain via direct mechanisms (i.e. reductions in lung volume or changes in leptin), it would be expected that greater reductions in weight would be associated with greater reductions in loop gain. However, no significant association was found between changes in loop gain and weight loss (r^2^ = 0.06, p= 0.112, see e-Figure 2*)*.


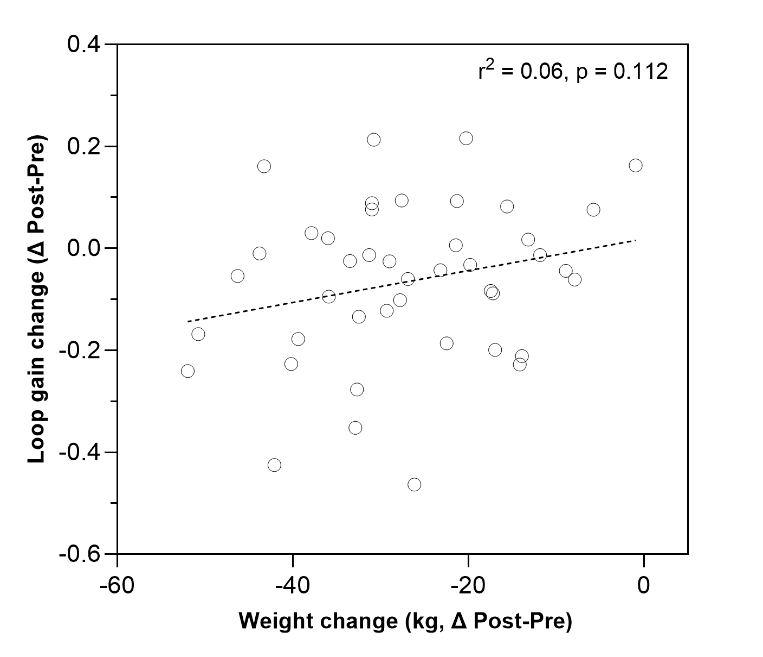


e-Figure 2: The relationship between change in weight and change in loop gain. There was no relationship between change in weight and change in loop gain.

Previous studies examining the impact of interventions that do not cause weight loss (e.g., CPAP^6^ and upper airway surgery^7–9^ on loop gain and OSA severity have demonstrated that reductions in loop gain tend to co-occur with reductions in OSA severity. These reductions in loop gain were attributed to reductions in OSA severity, specifically to reductions in the incidence and severity of hypoxic/hypoxemic stimuli, which may induce changes in chemo-responsiveness. Thus, in the current study, the observed reductions in loop gain may have been caused by reductions in OSA severity and, therefore, an indirect consequence of weight loss.

To further investigate whether the loop gain reductions in our study were associated with the decrease in OSA severity and not weight loss, we compared our study to three previous upper airway surgery studies^7–9^, see e-Table 9**.**

Upper airway surgery trials provide a good comparison measure because upper airway surgery is typically thought to decrease OSA severity by improving anatomy with no change in weight. Moreover, these trials used a similar pre-surgery and post-surgery study design and endotype measures as the current study. Notably, the average changes in AHI differed between studies, so we examined the changes in AHI and loop gain across responders and non-responders where possible.

***e-Table 9: Comparisons of Loop Gain and AHI Changes in previously published Upper Airway Surgery trials***

|  | Loop Gain | | | Total AHI (events/hr) | | |
| --- | --- | --- | --- | --- | --- | --- |
| Study | Before*^1^* | After*^1^* | Group  Change | Before*^1^* | After*^1^* | Group  Change |
| CURRENT STUDY | 0.65± 0.19 | 0.58± 0.19 | -0.07 | 43.4 [28.6, 64.8] | 16.9 [11.6, 33.3] | -26.5 |
| Responders | 0.57 [0.45, 0.68] | 0.52 ± 0.18 | -0.05 | 43.6 [36.0, 62.3] | 12.4 [6.9, 18.5] | -31.2 |
| Non-Responders | 0.65 [0.61, 0.77] | 0.65 ± 0.17 | 0 | 37.5 [25.1, 66.4] | 33.2 (16.9, 48.8) | -4.3 |
|  |  |  |  |  |  |  |
| Wong et al.^7^ | 0.45 ± 0.13 | 0.45 ± 0.12 | 0 | 38.7 [23.4, 79.2] | 22.0 [13.3, 53.5] | -16.7 |
| Responders | 0.43 [0.40, 0.56] | 0.40 [0.35, 0.42] | -0.03 | 55.8 ± 30.2 | 9.8 ± 6.3 | -46 |
| Non-Responders | 0.44 ± 0.14 | 0.48 ± 0.13 | 0.04 | 38.3 [22.1, 77.3] | 40.6[18.5, 65.8] | 2.3 |
|  |  |  |  |  |  |  |
| Joosten et al. ^9^ | 0.61 ± 0.02 | 0.58 ± 0.03 | -0.03 | 39.1 ± 4.2 | 26.5 ± 3.6 | -12.6 |
| Responders | 0.57 ± 0.04 | 0.48 ± 0.03 | -0.09 | 37.0 [23.5, 56.3] | 7.1 [3.2, 13.3] | -29.9 |
| Non-Responders | 0.65 ± 0.03 | 0.65 ± 0.03 | 0 | 24.2 [15.5, 53.8] | 17.2 [37.2, 52.3] | -7 |
|  |  |  |  |  |  |  |
| Li et al. 2019 ^8^ | 0.70 [0.58, 0.80] | 0.53 [0.46, 0.63] | -0.17 | 60.8 [33.7, 71.7] | 18.4 [9.9, 42.5] | -42.4 |

*^1^*Mean ± SD; Median [Interquartile Range]

Furthermore, in e-Figure 3 we assess the association between changes in loop gain and changes in AHI. We found a significant negative association, showing that a greater reduction in AHI was associated with a greater reduction in loop gain across studies (r^2^ =0.57, p=0.05). Notably, the changes in loop gain in the responder and non-responder groups are proportionally consistent with these surgical studies, which likely indicates that the reduction in loop gain observed in the current study could plausibly be unrelated to a change in weight.


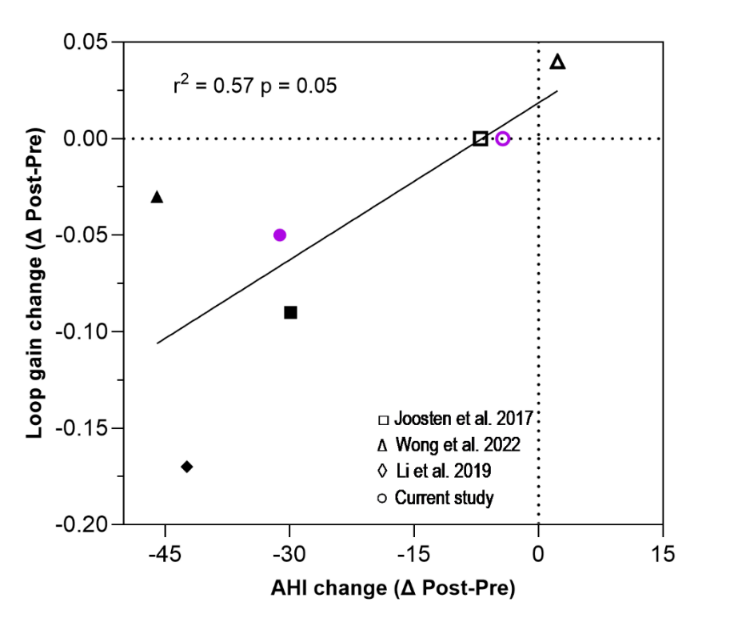


e-Figure 3: Relationship between Loop gain changes and AHI changes between previous upper airway surgery trials. The vertical axis shows changes in loop gain (post-surgery minus pre-surgery), and the horizontal axis shows changes in AHI (post-surgery minus pre-surgery). Data plotted show group average data from three previous trials assessing the impact of upper airway surgery on OSA endotypes. Specifically, squares show data from Joosten et al.^9^ triangles show data from Wong et al.^7^, and diamonds show data from Li et al.^8^. Data from the current study are shown in purple circles. To explore greater variance in the extent of change in AHI and loop gain, data from these studies is plotted separately for Responder (solid shapes) and non-responder groups (hollow shapes). These data show a significant association whereby greater average decreases in AHI are associated with greater average reductions in loop gain. Data from the current study (purple circles) fit this trend.

***SECTION 8: Comparison of the OSA Endotypes Derived from the Research PSG vs the Clinical PSG***

When we compared the values from of the key OSA endotypes derived from the clinical psg against the values from the research PSG, we saw that collapsibility measures (V_passive_ and V_active_) as well as arousal threshold were moderately-to-strongly associated with the variables when derived from the research PSG which employed the CPAP dial downs (see e-Figure 4). Loop gain values were not related to each other; which is likely due to the fact that we are comparing loop gain derived at different frequencies with each of these methods.


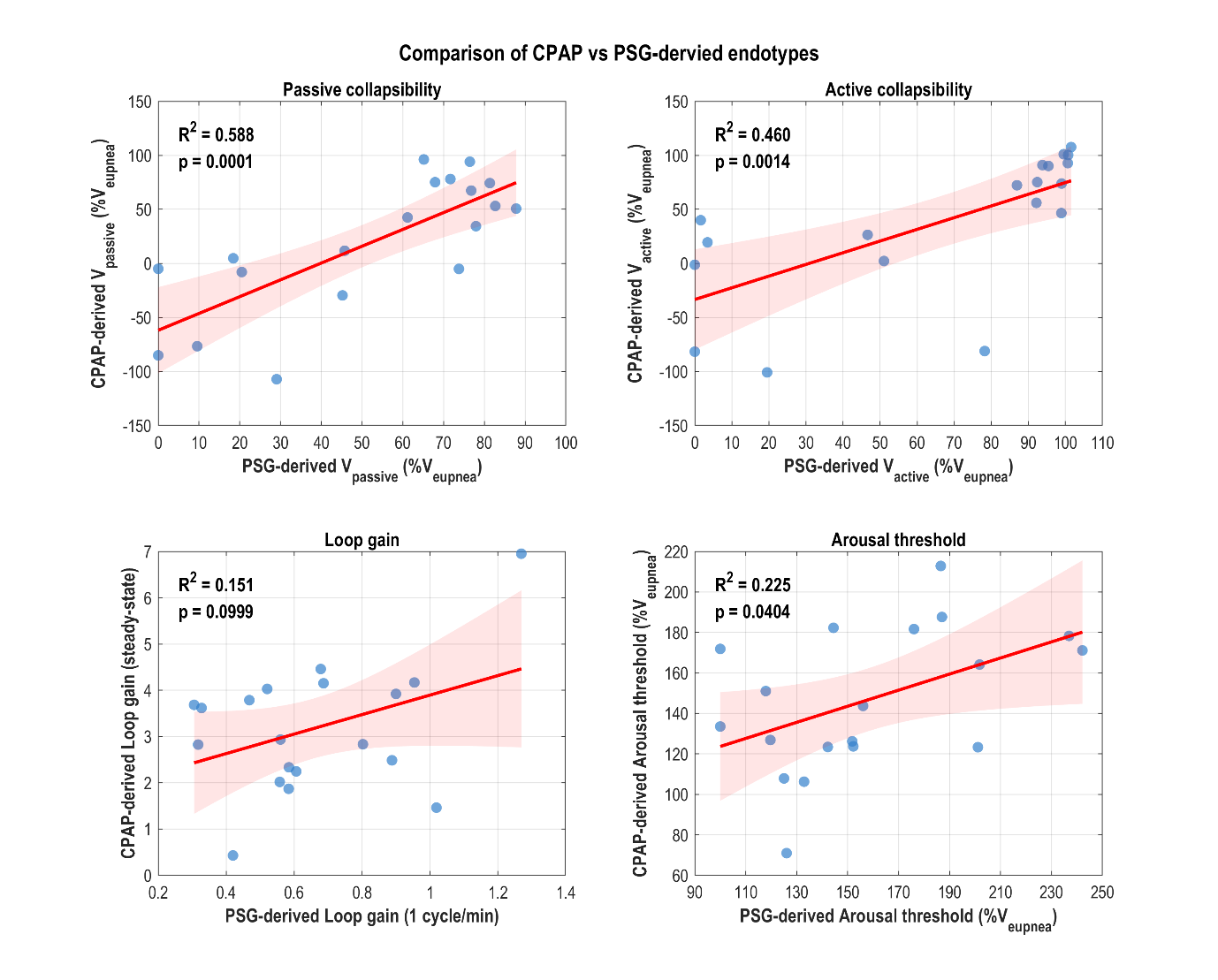
e-Figure 4: Endotypes from the research PSG (CPAP-derived, y-axes) were compared to those derived from the clinical PSG (PSG-derived, x-axes)**.** Note that the endotypes derived from the research PSG, are now expressed as a percentage of eupneic ventilation (V_eupnea_) for direct comparison against the PSG-derived values. Data from both baseline and follow-up are included on these graphs.

We also compared the standardized difference estimates (standardized betas) of the change in the OSA endotypes with both techniques (see e-Table 10 below).

**e-Table 10: Comparison of research PSG & clinical PSG standardized difference estimates**

|  | Research PSG  n=10 | Clinical PSG  n=43 |
| --- | --- | --- |
| Upper Airway Collapsibility *per V_passive_* | 0.428 | 0.307 |
| Loop Gain | -0.557 | -0.346 |
| Arousal Threshold | -0.412 | -0.241 |
| Muscle Compensation/Upper Airway Gain | -0.237 | -0.265 |

Across both datasets, the relative pattern of associations was consistent, with **loop gain showing the largest magnitude of change,** followed by **upper airway collapsibility (V_passive_),** and then arousal threshold. Muscle compensation (upper airway gain) demonstrated the smallest effect sizes in both groups. Although the beta estimates from research PSG-derived traits were generally of slightly greater magnitude compared to those from clinical PSG the overall interpretation remained similar, indicating that trait contributions to the outcome were directionally and proportionally stable across endotyping methods. It is important to note also that the larger standardized beta estimates observed in the research PSG group may partly reflect the smaller sample size (n = 10), which can increase sampling variability and lead to less stable effect estimates compared to the clinical PSG group (n = 43).

**References**

1. Bakker JP, Tavakkoli A, Rueschman M, et al. Gastric banding surgery versus continuous positive airway pressure for obstructive sleep apnea: A randomized controlled trial. *Am J Respir Crit Care Med*. 2018;197(8):1080-1083. doi:10.1164/rccm.201708-1637LE

2. Sutherland K, Smith G, Lowth AB, et al. The effect of surgical weight loss on upper airway fat in obstructive sleep apnoea. *Sleep and Breathing*. 2023;27(4):1333-1341. doi:10.1007/s11325-022-02734-8

3. Harris PA, Taylor R, Minor BL, et al. The REDCap consortium: Building an international community of software platform partners. *J Biomed Inform*. 2019;95:103208. doi:10.1016/J.JBI.2019.103208

4. Harris PA, Taylor R, Thielke R, Payne J, Gonzalez N, Conde JG. Research electronic data capture (REDCap)—A metadata-driven methodology and workflow process for providing translational research informatics support. *J Biomed Inform*. 2009;42(2):377-381. doi:10.1016/J.JBI.2008.08.010

5. Berry Brooks R. Gamaldo C.E. Harding S. M. Lloyd R. Marcus. C.L. Vaughn B.V. RB. *The AASM Manual for the Scoring of Sleep and Associated Events: Rules, Terminology and Technical Specifications, Version 2.0.3*. American Academy of Sleep Medicine; 2014.

6. Loewen A, Ostrowski M, Laprairie J, et al. Determinants of ventilatory instability in obstructive sleep apnea: Inherent or acquired? *Sleep*. 2009;32(10):1355-1365. doi:10.1093/sleep/32.10.1355

7. Wong AM, Landry SA, Joosten SA, et al. Examining the impact of multilevel upper airway surgery on the obstructive sleep apnoea endotypes and their utility in predicting surgical outcomes. *Respirology*. Published online October 1, 2022. doi:10.1111/resp.14280

8. Li Y, Ye J, Han D, et al. The effect of upper airway surgery on loop gain in obstructive sleep apnea. *Journal of Clinical Sleep Medicine*. 2019;15(6):907-913. doi:10.5664/jcsm.7848

9. Joosten SA, Leong P, Landry SA, et al. Loop Gain Predicts the Response to Upper Airway Surgery in Patients With Obstructive Sleep Apnea. *Sleep*. 2017;40(7). doi:10.1093/SLEEP/ZSX094

10. Strassberger C, Hedner J, Sands SA, et al. Night-to-Night Variability of Polysomnography-Derived Physiologic Endotypic Traits in Patients With Moderate to Severe OSA. *Chest*. 2023;163(5):1266-1278. doi:10.1016/j.chest.2022.12.029

11. Tolbert TM, Schoenholz RL, Parekh A, et al. Night-to-night reliability and agreement of obstructive sleep apnea pathophysiologic mechanisms estimated with phenotyping using polysomnography in cognitively normal elderly participants. *Sleep*. 2023;46(8). doi:10.1093/SLEEP/ZSAD058

12. Patil SP, Ayappa IA, Caples SM, John Kimoff R, Patel SR, Harrod CG. Treatment of adult obstructive sleep apnea with positive airway pressure: An American academy of sleep medicine clinical practice guideline. *Journal of Clinical Sleep Medicine*. 2019;15(2):335-343. doi:10.5664/jcsm.7640

13. Ramar K, Dort LC, Katz SG, et al. Clinical practice guideline for the treatment of obstructive sleep apnea and snoring with oral appliance therapy: An update for 2015. *Journal of Clinical Sleep Medicine*. 2015;11(7):773-828. doi:10.5664/jcsm.4858

14. Edwards BA, Thomson LDJ, Vena D, et al. Combined Supplemental Oxygen and Mandibular Advancement Device Therapy for Obstructive Sleep Apnea: A Randomized-Controlled Trial. *European Respiratory Journal*. Published online October 2, 2025:2501320. doi:10.1183/13993003.01320-2025

15. Wang X, Zhou T, Huang W, et al. Differences in Physiologic Endotypes Between Nonpositional and Positional OSA: Results From the Shanghai Sleep Health Study Cohort. *Chest*. 2024;166(1):212-225. doi:10.1016/j.chest.2024.01.021

16. Sands SA, Collet J, Gell LK, et al. Combination pharmacological therapy targeting multiple mechanisms of sleep apnoea: a randomised controlled cross-over trial. *Thorax*. 2024;79(3):259-268. doi:10.1136/THORAX-2023-220184

17. Edwards BA, Thomson LDJ, Vena D, et al. Combined Supplemental Oxygen and Mandibular Advancement Device Therapy for Obstructive Sleep Apnea: A Randomized-Controlled Trial. *European Respiratory Journal*. Published online October 2, 2025:2501320. doi:10.1183/13993003.01320-2025

18. Hoff E, Strassberger C, Zou D, Grote L, Stenlöf K, Hedner J. Modification of Endotypic Traits in OSA by the Carbonic Anhydrase Inhibitor Sulthiame. In: *Chest*. Vol 165. Elsevier Inc.; 2024:704-715. doi:10.1016/j.chest.2023.09.022

19. Hedner J, Stenlof K, Zou D, et al. A Randomized Controlled Clinical Trial Exploring Safety and Tolerability of Sulthiame in Sleep Apnea. *Am J Respir Crit Care Med*. 2022;205(12):1461-1469. doi:10.1164/RCCM.202109-2043OC
